# Formation of carbonatite-related giant rare-earth-element deposits by the recycling of marine sediments

**DOI:** 10.1038/srep10231

**Published:** 2015-06-02

**Authors:** Zengqian Hou, Yan Liu, Shihong Tian, Zhiming Yang, Yuling Xie

**Affiliations:** 1 State Key Laboratory of Continental Tectonics and Dynamics Institute of Geology, Chinese Academy of Geological Sciences, Beijing 100037, P. R. China; 2 Continental Tectonics Centre, Northwestern University, Xi’an, P.R. China; 3 Institute of Mineral Resources, Chinese Academy of Geological Sciences, Beijing 100037, P.R. China; 4 University of Science and Technology, Beijing, Beijing 100083, P. R. China

## Abstract

Carbonatite-associated rare-earth-element (REE) deposits are the most significant source of the world’s REEs; however, their genesis remains unclear. Here, we present new Sr-Nd-Pb and C-O isotopic data for Cenozoic carbonatite-hosted giant REE deposits in southwest China. These REE deposits are located along the western margin of the Yangtze Craton that experienced Proterozoic lithospheric accretion, and controlled by Cenozoic strike-slip faults related to Indo-Asian continental collision. The Cenozoic carbonatites were emplaced as stocks or dykes with associated syenites, and tend to be extremely enriched in Ba, Sr, and REEs and have high ^87^Sr/^86^Sr ratios (>0.7055). These carbonatites were likely formed by melting of the sub-continental lithospheric mantle (SCLM), which had been previously metasomatized by high-flux REE- and CO_2_-rich fluids derived from subducted marine sediments. The fertility of these carbonatites depends on the release of REEs from recycled marine sediments and on the intensity of metasomatic REE refertilization of the SCLM. We suggest that cratonic edges, particularly along ancient convergent margins, possess the optimal configuration for generating giant REE deposits; therefore, areas of metamorphic basement bounded or cut by translithospheric faults along cratonic edges have a high potential for such deposits.

Rare-earth elements (REEs) are essential for high-technology industries and crucial defense systems. China hosts one-third of the known REE reserves and produces 97% of the REE + Y^1^ in the world. A wide variety of REE deposits are found in China^2^,^3^, among which the carbonatite-associated REE deposits (CARDs), formed by REE-rich fluids exsolved from carbonatitic melts, are the most significant, accounting for approximately 65% of China’s REE reserves. The known CARDs include Bayan Obo (the world’s largest LREE-Fe-Nb deposit^4^), Maoniuping (the giant LREE deposit^5^) and numerous medium-large LREE deposits^6^,^7^, which are all located along cratonic margins (Fig. 1a) and share salient features, listed in Supplementary Table S1.

The Cenozoic Mianning-Dechang (MD) REE carbonatite belt in southwest China^8^ (Fig. 1b) is the best example of CARDs that developed along a cratonic edge; thus, it is an ideal location to test the hypothesis of REE-rich fertile carbonatites. The remnants of Proterozoic meta-volcanic arc rocks and arc granitoids (1000–740 Ma)^9^,^10^ exposed along the western margin of the Yangtze Craton suggest the subduction of a Proto-Tethyan oceanic lithosphere beneath the craton in the Neoproterozoic^9^,^10^,^11^,^12^. A series of Cenozoic strike-slip faults^8^ and extensive potassic (lamprophyre) magmatism, which peaked at ~35 Ma^13^ along the western margin of the craton (Fig. 1b), reflect overprinting and reworking associated with the Indo-Asian continental collision that began at 65 Ma^14^. The MD carbonatites occur as dykes and stocks associated with syenite intrusions that intruded Precambrian crystalline basement^15^. The spatial distribution of the carbonatites was controlled by Cenozoic strike-slip faults (Fig. 1b). The available age data indicate that the carbonatites and syenites were magmatically active from 40 to 12 Ma^15^,^16^, identical to the mineralization ages (34–11 Ma) of the CARDs in southwest China (Table S1)^17^. These deposits are genetically associated with carbonatitic dykes or stocks with fenitization alteration, and have a wide variety of mineralization styles with identical assemblages of bastnaesite + calcite + barite + fluorite^5^,^8^, varying from stockwork or vein systems (e.g., Maoniuping) and breccia pipe-hosted systems (e.g., Dalucao) to carbonatite stock systems (e.g., Lizhuang)^8^.

A previous study found that the MD carbonatites with CARDs have high radiogenic Sr isotopic compositions with (^87^Sr/^86^Sr)_i_ values >0.7055^15^, which are extremely rare among the world’s carbonatites^18^. The sources of the fertile carbonatite have been debate^18^,^19^,^20^,^21^, and it remains unclear whether the extreme REE enrichment of carbonatites as CARDs is caused primrily by intra-crustal processes or an anomalously REE-rich metasomatic mantle source. The answer to these questions bears on the origin of CARDs. Our new evidence suggests that the MD carbonatites were formed by melting of REE-refertilized metasomatic SCLM. The recycling of marine sediments, a process that introduces abundant LREEs into the SCLM via CO_2_-rich fluids during mantle metasomatism, is critical to the formation of CARDs.

## Results

We performed an integrated geochemical study of key samples from the MD carbonatites and CARDs in southwest China, including analyses of the chemical composition and Sr-Nd-Pb and C-O isotopic composition of the calcite separates and their host rocks. The major and trace element compositions and isotopic compositions are listed in Supplementary Tables S2–5 and presented in Figs. S1–6.

Based on the extensive petrographic study, the ratios of trace elements with similar geochemical behaviors (e.g., Sr/Ba, Rb/Sr, Nb/Y) and isotopic composition of Sr-Nd-Pb and C-O in the fresh bulk-rock samples and calcite separates (Tables S2–4) were used to characterize the MD carbonatites. The analyses show that the MD carbonatites have low SiO_2_ (<20.92%), FeO (<0.93%) and MgO (<1.50 %) concentrations and a wide range of CaO concentrations (21.41–55.4%) similar to that of calciocarbonatite^22^ (Fig. S1) but distinct from primary calcite carbonatites derived from direct melting of carbonated mantle sources^23^. These carbonatites have relatively low but variable concentrations of high field-strength elements (HFSEs; Fig. S2) with invariant Nb/Y ratios (1.0 ± 0.5; Fig. 2a). The carbonatites are extremely enriched in LREEs, with highly variable ratios of (La/Yb)_N_ (29–2889; Fig. S3), high concentrations of Sr (5100–56,300 ppm) and Ba (1070–60,800 ppm), relatively low ratios of Sr/Ba (Fig. 2b) and high ratios of Ba/Th (Fig. 2c). These features are consistent with other CARD-hosted carbonatites (e.g., Bayan Obo^24^,^25^, Mountain Pass^26^, and Laiwu^6^) but differ from most barren carbonatites worldwide (Figs. 2 and S4).

**Figure 1:**
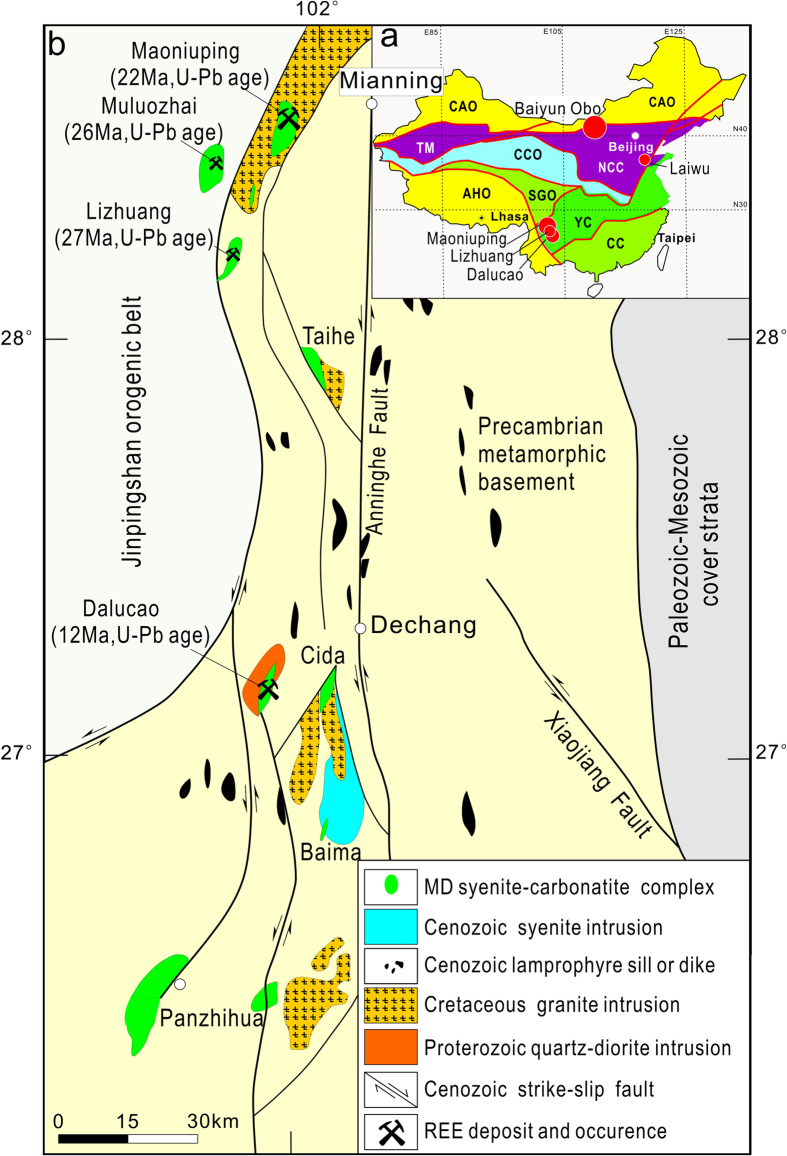
(**a**) Tectonic framework showing the spatial distribution of CARDs in China. (**b**) Sketch geological map showing the Cenozoic carbonatite–syenite complexes with zircon U-Pb ages (for syenite) in the MD REE belt, which is controlled by Cenozoic strike-slip faults. The Jinpingshan orogen was formed by subduction of the downgoing Proto-Tethyan oceanic lithosphere in the Proterozoic^11^,^12^. The Proterozoic metamorphic basement was locally covered by Paleozoic-Mesozoic sedimentary strata. NCC: North China Craton; TM: Tarim Block; YC: Yangtze Craton; CC: Cathaysia Craton; CAO: Central Asian Orogen; CCO: Central China Orogen; AHO: Alps-Himalayan Orogen; SGO: Songpan-Ganzi Orogen. The figure was generated using CorelDRAWX4 and the map will not have a copyright dispute.

A remarkable feature of the MD carbonatites is that their Sr-Nd isotope compositions are more radiogenic than the majority of global carbonatites with Sr-Nd isotopic ratios similar to that of ocean-island basalts (OIBs) involving high-μ U/Pb mantle (HIMU), enriched mantle I (EMI), and focal zone (FOZO) mantle components^27^,^28^,^29^,^30^,^31^. Both the bulk-rock samples and calcite separates yield Sr-Nd isotopic arrays shifted toward marine sediments (Fig. 3a) and away from East African carbonatite HIMU-EMI arrays^31^, and isotopically overlap with the Cenozoic REE-rich potassic rocks in southwest China^13^ (Fig. 3a). Such high radiogenic carbonatites are also found in other ore districts, e.g., the Bayan Obo^24^ and Laiwu^6^ (Figs. 1a and 3a).

This radiogenic signature is also demonstrated by the Pb isotopic compositions of the MD calcite separates and host carbonatites. Their compositions vary between EMI and EMII components but are shifted toward marine sediments^32^ (Fig. 3b; Fig. S5). The calcite separates have a wide range of ^207^Pb/^204^Pb (15.53–15.71) and ^208^Pb/^204^Pb (38.32–38.92) ratios^15^,^33^,^34^ that exhibit positive correlations with the ^87^Sr/^86^Sr values of the calcite separates (Table S3). However, the bulk-rock samples yield relatively uniform Pb isotopic compositions (Fig. 3b), which differ from most barren carbonatites because of relatively low ^206^Pb/^204^Pb ratios^27^,^28^ (Fig. S5). Twenty-three calcite separates and 22 fresh carbonatites yielded δ^18^O_V-SMOW_ values from 6.4% to 9.8% and δ^13^C_V-PDB_ values between −4.4% and −8.8%, which plot in the field of mantle-derived primary carbonatites^35^ (Fig. 3c). The samples from individual districts (e.g., Lizhuang and Dalucao) show distinct coherent trends with a weak positive correlation between the δ^18^O and δ^13^C values (Fig. 3c).

## Discussion

Carbonatites can become enriched in Sr, Ba, and REEs through intra-crustal processes, e.g., subsolidus remobilization^4^ and liquid immiscibility^25^,^26^. However, these processes do not explain the REE-Sr-Ba enrichment observed in the MD carbonatites. Our analyses indicate that the least-altered and freshest samples have extremely high concentrations of REEs (>2000 ppm), Sr (>5,000 ppm) and Ba (>1,000 ppm), although minor leaching of Sr and Ba from the MD carbonatites occurs by fenitization alteration. Experiments confirmed that liquid immiscibility can lead to strong partitioning of Sr, Ba, and REE into an immiscible carbonatite melts^36^,^37^; however, the MD syenites formed by immiscibility^15^ still yield high concentrations of Sr (up to 4,920 ppm), Ba (up to 7,663 ppm) and REEs (Fig. S3; Table S2). Using the partitioning coefficients of these elements in immiscible carbonatitic-silicate melts^36^,^37^, we estimated that their parent magmas have been extremely rich in Sr (>2000 ppm), Ba (>2000 ppm), and REEs (>5000 ppm), suggesting that such high concentrations may be a prerequisite for forming giant CARDs.

The high Sr and low Nd isotopic ratios in CARD-hosted carbonatites have been attributed to three processes: (1) crustal assimilation^15^, (2) sedimentary carbonate contamination^40^, and (3) heterogeneous mantle sources^6^,^15^,^24^. Crustal assimilation could elevate ^87^Sr/^86^Sr values and Rb/Sr ratios in carbonatites because of high Rb abundance^41^ and high ^87^Sr/^86^Sr ratios in the crust^42^. However, a positive correlation between ^87^Sr/^86^Sr and Rb/Sr ratios is not observed in the MD carbonatites (Fig. S6). The extremely high Sr concentrations (>5000 ppm) in the MD carbonatites may effectively buffer the effect of crustal assimilation on ^87^Sr/^86^Sr values^18^. Pb isotope is the most sensitive indictor for crustal assimilation^33^, however, the MD carbonatites yield relatively uniform Pb isotopic compositions (Fig. 3b), thus refuting the crustal assimilation hypothesis. Contamination by marine sediments with high ^87^Sr/^86^Sr ratios (>0.712)^42^ and high δ^13^C values (>−2%)^6^ could lead to a synchronous increase in ^87^Sr/^86^Sr, δ^18^O, and δ^13^C values in carbonatites (ref. 40). However, such contamination cannot explain why the Dalucao samples have the highest (^87^Sr/^86^Sr)_i_ values (Fig. 3a) and lowest δ^13^C values among the MD carbonatites (Fig. 3c). Although δ^13^C values are strongly temperature dependent (with heavier values found with decreasing temperatures of carbonatite formation^40^), the carbonatites from the three ore districts had similar formation temperatures^15^. Therefore, we contend that these highly radiogenic signatures reflect the heterogeneity of the mantle source. This argument is supported by two facts observed in the MD carbonatites: (1) a wide range of Li isotopic values (δ^7^Li: −4.5 to +10.8%) for carbonatite and calcite, which were probably produced by the mixture of a mantle component with subducted oceanic crust and marine sediments^43^; and (2) Sr-Nd-Pb isotopic compositions that overlap with those of associated Cenozoic potassic rocks, which were derived from a heterogeneous enriched mantle^13^ (Fig. 3a,b).

**Figure 2:**
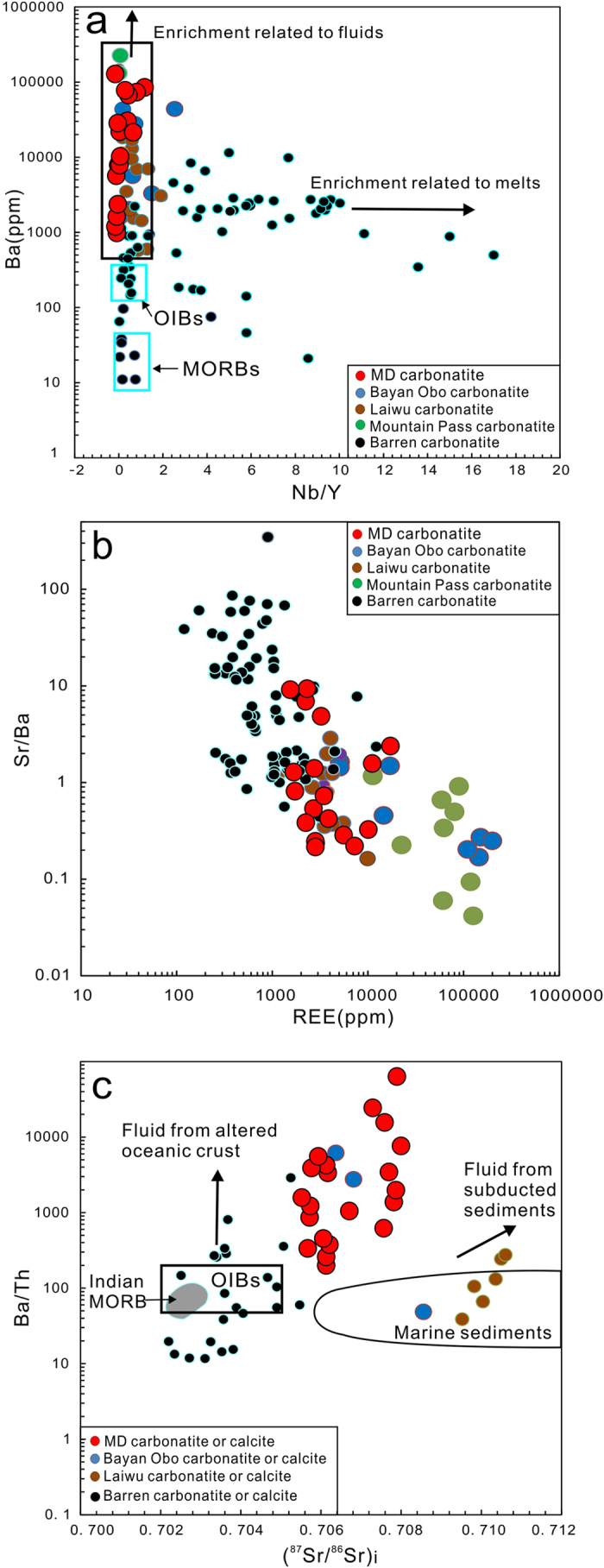
Plots of (**a**) Ba vs. Nb/Y, (**b**) Sr/Ba vs. REEs, and (**c**) Ba/Th vs. ^87^Sr/^86^Sr for the MD carbonatites and most global carbonatites. The fresh and least-altered carbonatite samples were plotted after eliminating altered and mineralized samples from the dataset (Supplementary Tables S2–5). (**a**) The majority of the CARD-hosted carbonatites have much lower Nb/Y ratios (1.0 ± 0.5) than most of the barren carbonatites, suggesting that their source enrichments are related to fluid metasomatism because fluid metasomatism does not strongly fractionate chemically similar elements, such as Nb and Y. Certain barren carbonatites have low Nb/Y ratios (≤1.0) and variable Ba concentrations (10–1000 ppm) close to that of ocean island basalts (OIBs) or mid-ocean ridge basalts (MORBs), implying derivation from a mantle plume source and/or interaction with lithospheric material during the generation of these magmas^18^. (**b**) The fresh samples show a weak negative correlation between Sr/Ba ratios and REE concentrations. (**c**) The much higher Ba/Th ratios of the MD carbonatites require the involvement of CO_2_-rich fluids derived from subducted sediments and oceanic crust. Certain carbonatites (e.g., Laiwu) have ^87^Sr/^86^Sr and Ba/Th ratios that overlap with those of marine sediments, suggesting the involvement of subducted sediments during carbonatite formation^6^. All data from this study (Tables S2–3) and Ying *et al.* (2004) and Yang *et al.* (2011).

**Figure 3:**
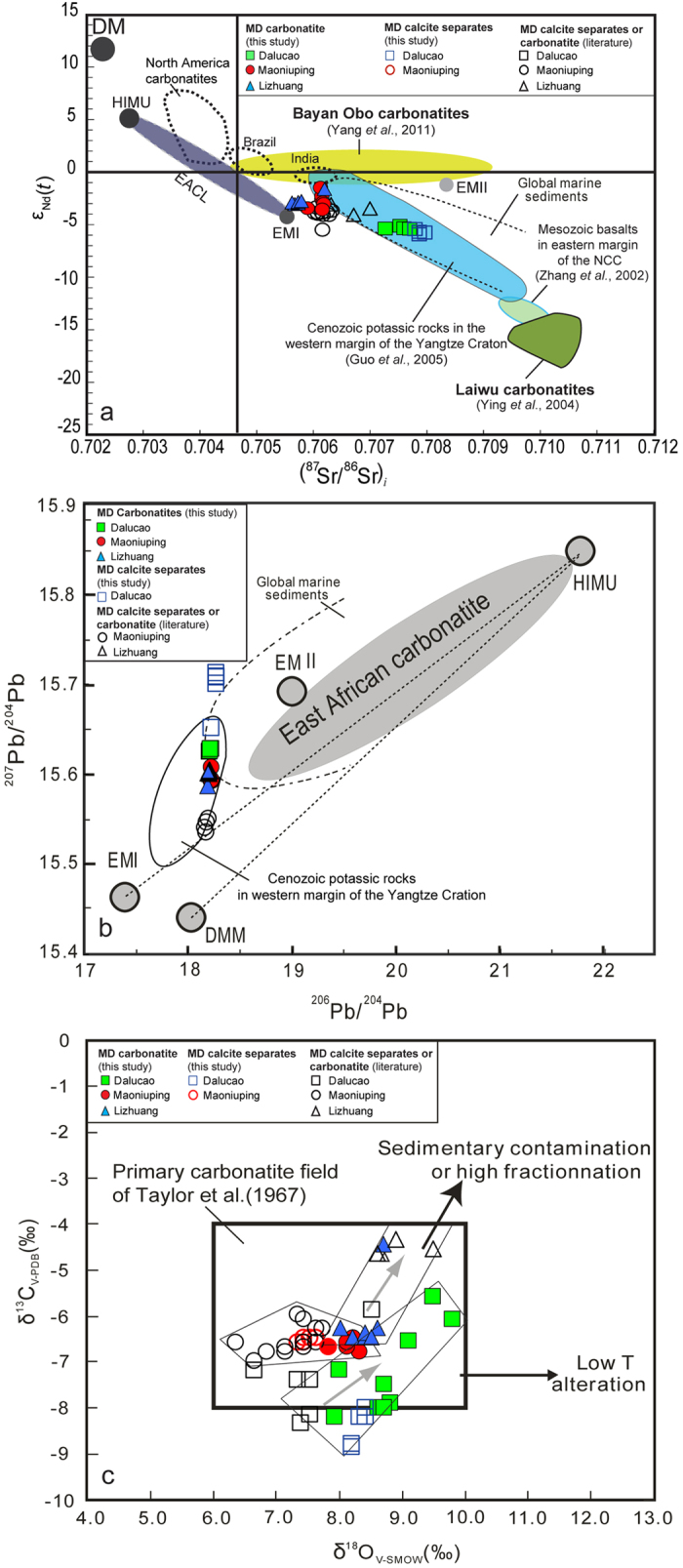
(**a**) Sr–Nd isotopic compositions of the fresh MD carbonatites and their calcite separates. Other CARD-hosted carbonatites (e.g., Bayan Obo^24^ and Laiwu^6^) and associated Cenozoic-Mesozoic igneous rocks in China^13^,^38^ are shown for comparison with carbonatites from southwest China, East Africa (EACL)^27^, North America^26^, Brazil^22^ and India^29^. The dotted line outlines the global marine sediment field^32^. The MD samples yield an Sr-Nd isotopic array shifted toward marine sediment. The DM (depleted mantle), HIMU (high-μ mantle), EMI (enriched mantle I), and EMII (enriched mantle II) fields are distinct mantle end-members^39^. (**b**) ^207^Pb/^204^Pb vs. ^206^Pb/^204^Pb correlation diagram for the fresh MD carbonatites and calcite separates. Carbonatites from East Africa^31^ and Cenozoic potassic rocks from southwest China^13^ are shown for comparison. (**c**) δ^13^C vs. δ^18^O correlation diagram for the calcite separates and fresh carbonatites. Previously collected calcite data were re-examined by extensive microscope observations and plotted for comparison with the fresh carbonatites. All the plotted data fall into the fields of mantle-derived primary carbonatites^35^. Fresh samples from the Dalucao and Lizhang districts feature two distinct coherent trends with weak positive correlations between δ^18^O_VSMOW_ and δ^13^C_VPDB_ values. All data from Tables S2–4.

Previous studies indicate that most of the world’s carbonatites usually have Sr-Nd-Pb isotope ratios similar to that of OIBs. Thus, these carbonatites are widely regarded to be derived from the melting of sub-lithospheric mantle triggered by either asthenospheric upwelling or mantle plume activity^18^. However, this model cannot explain the Sr-Nd-Pb isotopic arrays for the MD carbonatites (Fig. 3) or extreme enrichment of Sr, Ba, and REEs (Fig. 2). Our data suggest that the MD carbonatites were derived from a metasomatic-enriched SCLM with involvement of subducted marine sediments, which is consistent with modeling results for the Sr-Nd isotopic data^15^ and Li isotopic data^43^.

The metasomatism of fluids derived from the subducted oceanic slab (i.e., MORB) is capable of hydrating, oxidizing, and refertilizing the SCLM with ore-forming elements^44^,^45^,^46^, but it would be difficult for this process to sufficiently carbonate the mantle to generate carbonatite melts^18^, thus requiring the recycling of marine sediments into the mantle source^47^,^48^. Direct melting of the recycled carbonate sediments would produce primary carbonatitic melts with more radiogenic Sr-Nd isotopic compositions (^87^Sr/^86^Sr >0.706)^48^ and relatively low Ba/Th ratios (<200)^49^. Compared with such carbonatitic melts, the MD carbonatites have similar ^87^Sr/^86^Sr values (0.705–0.708) but unusually high Ba/Th ratios (>200), suggesting the involvement of CO_2_-rich fluids from the recycled marine sediments (Fig. 2c).

CO_2_-rich fluids generally have high concentrations of large-ion-lithophile elements (LILE) and high LILE/HFSE ratios, which is primarily a result of the high mobility of LILEs and relative insolubility of HFSEs and Y in aqueous fluids^50^,^51^. The SCLM metasomatism by such CO_2_-rich fluids would produce a carbonated peridotite refertilized with respect to the LILEs (e.g., Ba and Sr). Subsequent melting of this carbonated peridotite would concentrate the alkaline-earth elements (e.g., Ca, Sr, and Ba) into the carbonated melt^52^,^53^,^54^; the HFSEs (Nb, Ta, Zr, and Hf), heavy REEs and Y would be retained in Ti-oxides because of their high D_min/melt_ values^55^. This scenario is consistent with certain geochemical features of the MD carbonatites, such as their extreme enrichment in Sr and Ba, relative depletion in HFSEs, and low ratios of Nb/Y (Fig. 2c).

Experiments indicate that CO_2_-rich fluids could transport and add LREEs into metasomatized SCLM at pressures of >25 kbar^56^ because of the high partitioning coefficients of LREEs in CO_2_-rich fluids/melts^54^, thus explaining the common REE enrichments in most of the world’s carbonatites^18^. However, the extremely high REE concentrations in the MD carbonatites indicate that LREE enrichments can be controlled by additional factors, including variations in the amount of metasomatically introduced components, phases of subducted sediments, and total volatile concentrations within the magmatic source^37^.

Research on subducting sediments indicates that REE and Ba abundances are closely linked to seafloor hydrothermal sediments^1^ and that Sr contents are controlled by carbonate phase abundances^32^. Recent observations also demonstrate that REEs are strongly concentrated in metalliferous, Fe-oxyhydroxide-bearing sedimentary muds on the seafloor, such as in the eastern part of the southern Pacific (>1000 ppm total REE + Y) and northern Pacific (>400 ppm total REE + Y)^1^. These concentrations are in turn diluted by increases in the proportion of carbonate phases within the marine sediments^1^, which indicates that REE concentrations in subducted sediments are controlled by the ratio of hydrothermal mud to carbonate phases. As a first-order approximation, the Sr/Ba ratio of marine sediments may reflect the quantitative proportions of carbonate and hydrothermal phases and amount of REEs within the sediments. This relationship predicts that carbonatitic melts derived from carbonated mantle metasomatized by CO_2_-rich fluids from recycled marine sediments should have Sr/Ba ratios similar to those of the subducted sediments because of the similar geochemical behaviors of Sr and Ba. The negative correlation found between Sr/Ba ratios and REE concentrations in global carbonatites (Fig. 2a) also suggests that the carbonate/hydrothermal phase proportion in the subducted sediments exerts important control on the REE concentration of carbonatite melts. The proportion of these two phases probably controls the quantity of REEs released into the CO_2_-rich fluids, thereby controlling the quantity of REEs that is metasomatically introduced into the SCLM. Higher proportions of hydrothermal phases in the subducted sediments increases the amount of LREEs that are introduced into the SCLM; and the higher fluxes of LREE- and CO_2_-rich fluids during metasomatism of the SCLM, the higher concentration of REEs in the carbonatite melts. We therefore conclude that giant CARDs form from LREE-rich carbonatitic melts derived from REE-refertilized mantle sources related to the recycling of REE-rich marine sediments.

This recycling of subducted marine sediments for CARD formation is consistent with the continuous subduction of a Proto-Tethyan oceanic lithosphere beneath the Yangtze Craton in the Neoproterozoic^9^,^10^,^11^,^12^ and development of a 1000-km long, Neoproterozoic magmatic arc along the western margin of the Yangtze Craton^10^. The Nd model ages for the MD carbonatites (0.69–1.63 Ga; Table S3) and associated Cenozoic potassic rocks (0.54–1.60 Ga)^13^ suggest that the enrichment of SCLM metasomatized by subducted slab fluids was likely multi-phase, with most of the enrichment clustering in the Neoproterozoic. Similar metasomatic enrichment has also been recognized in the Bayan Obo and Laiwu districts, where an Archean-depleted SCLM was metasomatized by subducted sediments at 1.9–2.0 Ga^57^ and a Paleozoic-enriched SCLM was replaced by EMII-type mantle because of subduction of the paleo-Pacific plate since the Late Jurassic^38^, respectively.

**Figure 4:**
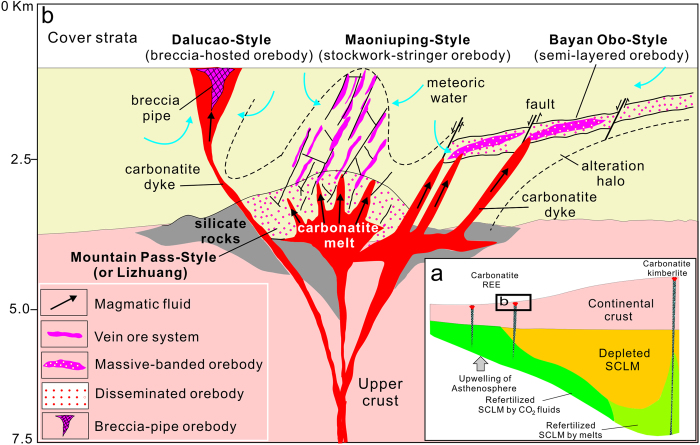
Metasomatic refertilization of the SCLM and formation of fertile carbonatites and CARDs. (**a**) Upwelling of asthenosphere triggers the melting of refertilized SCLM^46^ that was previously metasomatized by CO_2_-rich fluids derived from marine sediments associated with “fossil” subduction zones. The subducted sediments released their REEs into CO_2_-rich fluids that metasomatized old depleted or enriched SCLM to form an unusually REE-rich, carbonated mantle source, which then produced carbonatite melts or CO_2_-rich silicate melts. The margins of the craton experience low degrees of partial melting, and the melts ascend through fracture zones into the overriding crust. (**b**) Schematic illustration of models of CARD formation, including a variety of orebodies formed by fluids exsolved from REE-rich carbonatitic magmas emplaced at shallow crustal levels. Lateral migration, replacement, open-space filling, and focused discharges of ore-forming fluids produced semi-stratabound (Bayan Obo-style)^24^,^25^, disseminated (Lizhuang or Mountain Pass-style)^8^,^26^,^58^, stringer-stockwork (Maoniuping-style)^5^ and breccia pipe (Dalucao-style)^8^ orebodies with associated fenitization and K-silicate alterations, respectively.

Metasomatic refertilization of metals (Cu, Au, and REEs) occurred in the SCLM with the intensity increasing toward cratonic margins above ancient subduction zones, which created lateral compositional heterogeneity^46^ (Fig. 4a). The formation of fertile carbonatites requires a thick lithosphere and/or high pressure (>25 kbar)^56^, metasomatic enriched mantle sources^15^, and favorable pathways for magma ascending into the overlying crust^46^ where REE-rich fluids exsolve from the cooling magma^26^,^58^. The optimal configuration of all three factors only occurs along the margins of a craton with a continental root, rather than in a modern subduction zone with relatively thin lithosphere. The melting of REE-refertilized mantle was triggered by upwelling of asthenosphere^59^, and the resulting magmas ascended along translithospheric faults along cratonic edges and emplaced at shallow crust (Fig. 4a). These magmas rapidly exsolved highly oxidized, REE-rich fluids during stress relaxation^58^, which led to fenitization around the carbonatite stocks^60^ and precipitation of bastnaesite when the temperatures decreased, thus forming CARDs with distinct styles of mineralization^8^ (Fig. 4b). These patterns suggest that areas of metamorphic basement bounded or cut by translithospheric faults along cratonic margins and other lithospheric boundaries have significant potential for such REE deposits.

## Methods

### Whole-rock chemical analysis

All the samples were partially analyzed at the National Research Center of Geoanalysis, CAGS, Beijing. Whole-rock powders were weighed (0.7 g) and then mixed with 5.3 Li_2_B_4_O_7_, 0.4 g LiF and 0.3 g NH_4_NO_3_ in a 25 ml porcelain crucible. The mixture powder was then transferred to a platinum alloy crucible, and 1 mL LiBr solution was added to the crucible and then dried. The sample was then melted in an automatic flame fusion machine and the resulting cooled glass was used for the X-ray ﬂuorescence (XRF) major element analyses. Analytical errors were <2 relative %. The analytical errors were less than 2%. For the trace element (and REE) analyses, whole-rock powders were weighed (50 mg) and then dissolved in 1 mL distilled HF and 0.5 mL HNO_3_ in 15 ml Savillex Teflon screw-cap capsules at 190 °C for 1 day, dried, and then digested with 0.5 mL HNO_3_ and dried again. The capsules were digested with 0.5 mL HNO_3_ and dried again. The samples were then digested with 5 mL HNO_3_ and sealed at 130 °C for 3 hours. After cooling, the solutions were transferred to plastic bottles and diluted to 50 ml before analysis. The sample solutions were analyzed for trace elements via inductively coupled plasma mass spectrometry (ICP-MS). The analytical precision for most elements was greater than 5%.

### C and O isotopic analysis

The C and O isotopic compositions of carbonates were partially analyzed via the GasBench II method on a MAT253 mass spectrometer in the Institute of Mineral Resources, CAGS (Beijing). Approximately 0.1 mg sample powder was added to a reaction bottle with a total volume of 12 ml. Less than eighty-eight samples, including eighteen Chinese isotopic standards (GBW04416, GBW04417, GBW04405 and GBW04416), were prepared for every measurement. The sample powders were flushed for 600 s with pure He (99.999%) at a flow rate of 100 ml/min. Each sample was then dissolved with 5 drops of 100% anhydrous H_3_PO_4_ at 72 °C for more than four hours to liberate CO_2_ from the calcite. The CO_2_ was injected into a loop, separated via a chromatographic column (Poraplot Q, 25 m * 0.32 mm, Varian Ltd) at a constant 70 °C, dried using a Nafion membrane, and analyzed by an MAT 253 mass spectrometer. The analytical results of eighteen isotopic standards indicate that the external precision was better than 0.10% for the δ^13^C and δ^18^O values. The δ^13^C and δ^18^O results were reported in standard delta notation (% units) relative to the Vienna Pee Dee Belemnite (VPDB).

### Sr, Nd, and Pb isotopic analysis

The Sr-Nd-Pb isotope compositions were partially analyzed at the Laboratory of Isotope Geology, CAGS (Beijing). For the Rb-Sr and Sm-Nd isotope analyses, rock chips of less than 20 mesh size were used. The sample powders were spiked with mixed isotope tracers and then dissolved in Teflon capsules with HF + HNO_3_. After the samples were fully dissolved and dried to remove HF, the samples were dissolved with HCl. These samples were then dissolved with HClO_4_ and transferred to an HCl medium. The Sr and REE fractions were separated in solution using cationic ion-exchangers (AG 50 W(H^+^), 200 to 400 mesh) in columns. Then, Sr and Nd were collected in the proper sequence via leaching with 4 mol ml^−1^ HCl. The Sr fraction was purified again according to the above procedure for complete separation. Then, the solution was dried for isotopic measurement. The collected Nd fraction was evaporated and dissolved in 0.2 mol ml^−1^ HCl, and the Nd was separated from the REE fractions using cationic ion-exchange columns and extraction and eluviation resin (HDEHP). The solution was then rinsed with 0.2 mol ml^−1^ HCl to remove Sm, and Sr isotopic measurements were performed on an MAT 262 mass spectrometer. The mass fractionation corrections for the Sr isotopic ratios were based on ^88^Sr/^86^Sr = 8.37521. The international standard NBS987 yielded ^87^Sr/^86^Sr = 0.710250 ± 10 (2σ). The Nd isotopic measurement was performed on a Nu Plasma HRMC-ICP-MS (Nu Instruments) 262 mass spectrometer. The mass fractionation corrections were based on ^146^Nd/^144^Nd = 0.7219. The international standard is JMC Nd_2_O_3_, in which ^143^Nd/^144^Nd = 0.511125 ± 10 (2σ). The entire procedure blank was less than 10^−9^–10^−9^ g for Sr and 5 × 10^−11^ g for Nd. The analytical errors are given as 2σ. The ^87^Rb/^86^Sr and ^147^Sm/^144^Nd ratios were calculated using the Rb, Sr, Sm and Nd concentrations, and the initial ^87^Rb/^86^Sr and ^143^Sm/^144^Nd ratios were calculated using SHRIMP (sensitive high-resolution microprobe) concordant ages. Initial εNd values and ^87^Sr/^86^Sr ratios were calculated according to the ages of the corresponding carbonatites or syenites.

The measurements of the Pb isotopes were performed with a Nu Instrument multi-collector ICP-MS using the method described by Belshawa *et al.* (1998)^61^. The samples were digested using a mixture of ultrapure HF and HNO_3_ at 800 °C for 72 h, followed by purification using conventional ion-exchange chromatography (AG1X8, 200–400 resin). A portion of the sample (0.2 ml) was added and drip washed with 1 ml of 1 mol/L HBr as eluant five times and 0.5 ml of 2 mol/L HBr as eluant once. The Pb was collected by drip washing with 1 ml of 6 mol/L HCl and 0.5 ml of 6 mol/LHCl . The entire procedure blank is less than 0.1 ng. During the period of analysis, repeat analyses of the international standard NBS981 yielded the following values: ^208^Pb/^206^Pb = 2.1674 ± 0.0004; ^207^Pb/^206^Pb = 0.91478 ± 0.00018; ^206^Pb/^204^Pb = 16.9402 ± 0.0070; ^207^Pb/^204^Pb = 15.4966 ± 0.0030; and ^208^Pb/^204^Pb = 36.7155 ± 0.0120 (2σ).

## Additional Information

**How to cite this article**: Hou, Z. *et al.* Formation of carbonatite-related giant rare earth element deposits by the recycling of marine sediments. *Sci. Rep.* **5**, 10231; doi: 10.1038/srep10231 (2015).

## Supplementary Material

Supplementary Information
