# Supplementary material for: Formation of carbonatite-related giant rare-earth-element deposits by the recycling of marine sediments
Source: Sci Rep. 2015 Jun 2;5:10231. doi: 10.1038/srep10231 (PMC4451788; doi:10.1038/srep10231)
Supplement: Supplementary Information [file srep10231-s1.pdf]

Supplementary Information

Formation of carbonatite-related giant rare-earth-element deposits by the recycling of marine sediments

Zengqian Hou[1,2*], Yan Liu[1], Shihong Tian[3], Zhiming Yang[1], Yuling Xie[4]

1. Institute of Geology, CAGS, Beijing 100037, P. R. China
2. Continental Tectonics Centre, Northwestern University, Xi'an, P.R. China
3. Institute of Mineral Resources, CAGS, Beijing 100037, P.R. China
4. Beijing University of Science and Technology, Beijing 100082, P. R. China

*Corresponding author: Hou Zengqian

E-mail address: Houzengqian@163.com

Supplementary Materials

Table S1. The salien features of major CARDs in China

Table S2. Summary of major and trace element compositions of the fresh or least-altered MD carbonatites in SW China.

Table S3. Sr-Nd-Pb isotopic compositions of the fresh or least-altered MD carbonatites and their calcite separates in SW China.

Table S4. C–O isotopic compositions of the fresh or least-altered MD carbonatites and their calcite separates in SW China.

Table S5. Major and trace element compositions of representative samples of fresh or least-altered carbonatites worldwide

Fig. S1. Carbonatite classification plot of CaO-MgO-FeO+Fe_2O_3+MnO (Woolley, 1989)[32]. The MD carbonatites have chemical compositions similar to calciocarbonatite. All data from Table S2.

Fig. S2. Primitive mantle-normalized trace element variation diagrams for the MD carbonatites (**a**) and associated syenites (**b**) in the Cenozoic carbonatite-syenite complexes, SW China. All data from Table S2.

Fig. S3. REE patterns of the MD carbonatites and associated syenites in the Southwestern China. All data from Table S2.

Fig. S4. Plots of REEs vs. CaO/MgO (**a**), REEs vs. total FeO/MgO(**b**), and (c) REEs vs. Ba (**c**) and REEs vs. Sr (**d**) for the MD carbonatites and other carbonatites workdwide. Only the least-altered or fresh samples are plotted for comparison. These data indicate that REE enrichment is unrelated to both type of carbonatite and composition of major elements, but show a weak positive correlation with Sr and Ba contents of carbonatites. All data from Tables S2 and S5.

Fig. S5. $^{208}Pb/^{204}Pb$ vs $^{206}Pb/^{204}Pb$ (**a**), $(^{87}Sr/^{86}Sr)_i$ vs $^{206}Pb/^{204}Pb$ (**b**) and $^{143}Nd/^{144}Nd$ vs $^{206}Pb/^{204}Pb$ (**c**) correlation diagrams for the MD carbonatites and their calcite separates. The dotted line outlines the field of global marine sediments[33]. Most of the MD carbonatites and calcites show a linear array broadly plotting between EM I and EMII, but shifting toward marine sediments. Carbonatites from East Africa[34-35] and India[36] and Cenozoic potassic rocks[37] are shown for comparison. EMI, EMII, HIMU, FOZO, DM mantle components[38-40] are shown for reference.

Fig. S6. Plot of $^{87}Sr/^{86}Sr$ vs. Rb/Sr ratios for the MD carbonatites. The least-altered carbonatites from the Bayan Obo[10] and Laiwu districts[8] in China are shown for reference. The lack of a positive correlation for the MD carbonatites suggests that they did not assimilate significant amounts of crustal materials with high Rb/Sr ratios. Data from Tables S2-3.

Reference

1. Zhang, Z-Q., Yuan, Z-X., Tang, S-H., Bai, G & Wang, J-H. Age and geochemistry of the Bayan Obo Ore deposits. Geological Publishing House: Beijing, 1-222 (2003)

2. Yang, X-Y., Sun, W-D., Zhang Y-X.& Zheng, Y-F. Geochemical constraints on the genesis of the Bayan Obo Fe–Nb–REE deposit in Inner Mongolia, China. *Geochim Cosmochim Ac* **73,** 1417–1435 (2009)

3. Ling, M-X. et al. Formation of the world's largest REE deposit through protracted fluxing of carbonatite by subduction-derived fluids. *Sci Rep* **3**, 1-8 (2013)

4. Yuan, Z.X. et al. The Maoniuping rare earth ore deposit, Mianning county, Sichuan Province. Seismological Press: Beijing 150p (in Chinese) (1995)

5. Hou, Z.Q. et al. The Himalayan Mianning-Dechang REE belt associated with carbonatite-alkalinc complex in the eastern Indo-Asian collision zone, SW China. *Ore Geol Rev* **36**, 65-89 (2009)

6. Niu, H.-C. & Lin, C.-X. Genesis of the REE ore deposit in Mianning County, Sichuan Province, China. *Mineral Deposits* **13**, 345-353 (in Chinese with English abstract) (1994)

7. Yang, Y.H. et al. In situ U-Pb dating of bastnaesite by LA-ICP-MS. *J Anal Atom Spectrom* **29**, 1017-1023 (2014)

8. Ying, J-F., Zhou, X-H. & Zhang H-F. Geochemical and isotopic investigation of the Laiwu–Zibo carbonatites from western Shandong Province, China, and implications for their petrogenesis and enriched mantle source. *Lithos* **75**, 413– 426 (2004)

9. Hou, Z.Q. et al. The Himalayan collision zone carbonatites in western Sichuan, SW China: Petrogenesis, mantle source and tectonic implication. *Earth Planet Sc Lett* **244**, 234–250 (2006)

10. Yang, K.-F., Fan H.-R., Santosh, M., Hu, F.-F. & Wang, K.-Y. Mesoproterozoic carbonatitic magmatism in the Bayan Obo deposit, Inner Mongolia, North China: Constraints for the mechanism of super accumulation of rare earth elements. *Ore Geol Rev* **40**, 122–131 (2011)

11. Xu, C. et al. Geochemistry of carbonatites in Maoniuping REE deposit, Sichuan Province, China. *Sci China Earth Sci* **32,** 635–643 (2002)

12. Wang, D.H. et al. A special orogenic-type rare earth element deposit in Maoniuping, Sichuan, China: geology and geochemistry. *Resour Geol* **15,** 177–188 (2001)

13. Sun, J., Zhu, X.H., Chen, Y.L. & Fang, N. Iron isotopic constraints on the genesis of Bayan Obo ore deposit, Inner Mongolia, China. *Precambrian Res* **235**, 88–106 (2013)

14. Yang, K.F., Fan, H.R., Santosh, M., Hu, F.F. & Wang, K.Y. Mesoproterozoic carbonatitic magmatism in

the Bayan Obo deposit, Inner Mongolia, North China: Constraints for the mechanism of super accumulation of rare earth elements. *Ore Geol Rev* **40**, 122–131 (2011)

15. Yang, K.F. Fan, H.R., Santosh, M., Hu, F.F. & Wang, K.Y. Mesoproterozoic mafic and carbonatitic dykes from the northern margin of the North China Craton: Implications for the final breakup of Columbia supercontinent. *Tectonophysics* **498**, 1–10 (2011)

16. Castor, S. B. The Mountain Pass rare-earth carbonatite and associated ultrapotassic rocks, California. *Can Mineral* **46,** 779–806 (2008)

17. Andrade, F.R.D., Möller, P., Luders, V., Dulski, P. & Gilg, H.A. Hydrothermal rare earth elements mineralization in the Barra do Itapirapuã carbonatite, southern Brazil: behavior of selected trace elements and stable isotopes C, O. *Chem Geol* **155**, 91–113 (1999)

18. Le Roex. A.P. & Lanyon, R. Isotope and trace element geochemistry of cretaceous damaraland lamprophyres and carbonatites, northwestern Namibia: Evidence for Plume–Lithosphere Interactions. *J Petrol* **39,** 1117–1146 (1998)

19. Ying, J.F., Zhou, X.H. & Zhang, H.F. Geochemical and isotopic investigation of the Laiwu–Zibo carbonatites from western Shandong Province, China, and implications for their petrogenesis and enriched mantle source. *Lithos* **75,** 413–426 (2004)

20. Simonetti, A. & Bell, K. Nd, Sr and Pb isotopic data from the Napak carbonatite-Nephelinite center, eastern Uganda: an example of open-system crystal fractionation. *Contrib Mineral Petrol* **115,** 356–366 (1994)

21. Srivastava, R. K. Petrology, geochemistry and genesis of rift-related carbonatites of Ambadungar, India. *Mineral Petrol* **61**, 47–66 (1997)

22. Zaitsev, A.N. Wall, F & Le Bas M. J. REE-Sr-Ba minerals from the Khibina carbonatites, Kola Peninsula, Russia: their mineralogy, paragenesis and evolution. *Mineral Mag* **62**, 225–250 (1998)

23. Verhulst, A., Balaganskaya, E., Kirnarsky, Y & Demaiffe, D. Petrological and geochemical (trace elements and Sr-Nd isotopes) characteristics of the Paleozoic Kovodor ultramafic, alkaline and carbonatite intrusion (Kola Peninsula, NW Russia). *Lithos* **51**, 1–25 (2000)

24. Smithies, R.H & Marsh, J.S. The Marinkas Quellen carbonatite complex, southern Namibia: carbonatite magmatism with uncontaminated depleted mantle signature in a continental setting. *Chem Geol* **148**, 201–212 (1998)

25. Thompson, R.N., Smith, P.M., Gibson, S.A., Mattey, D.P & Dickin, A.P. Ankerite carbonatite from Swartbooisdrif, Namibia: the first evidence for magmatic ferrocarbonatite. *Contrib Mineral Petrol* **143**, 377–395 (2002)

26. Brassinnesa, S., Balaganskayab, E. & Demaiffea, D. Magmatic evolution of the differentiated ultramafic, alkaline and carbonatite intrusion of Vuoriyarvi (Kola Peninsula, Russia). A LA-ICP-MS study of apatite. *Lithos* **85**, 76–92 (2005)

27. Hubberten, H.-W., Katz-Lehnert, K. & Keller, J. Carbon and oxygen isotope investigations in carbonatites and related rocks from the Kaiserstuhl, Germany. *Chem Geol* **70**, 257–274 (1988)

28. Secher, K& Larsen, L.M.Geology and mineralogy of Sarfartoq carbonatite complex, southern West Greenl and. *Lithos* **13**, 199–212 (1980)

29. Dunworth, E.A & Bell, K. The Turiy Massif, Kola Peninsula, Russia: isotopic and geochemical evidence for multi-source evolution. *J Petrol* **42**, 377–405 (2001)

30. Giovanni, M. et al. Trace element geochemistry of the Mt Vulture carbonatites, southern Italy. *Int Geol Rev* **55**, 1541–1552 (2013)

31. Ebya, G. N., Lloydb, F.E& Woolleyc, A.R. Geochemistry and petrogenesis of the Fort Portal, Uganda, ext rusive carbonatite. *Lithos* **113**, 785–800 (2009)

32. Woolley, A.R. The spatial and temporal distribution of carbonatites. In: *Carbonatites Genesis and Evolution.* 15–37 (Unwin Hyman, 1989)

33. Plank, T. & Langmuir, C. H. The chemical composition of subducting sediment and its consequences for the crust and mantle. *Chem Geol* **145**, 325–394 (1998)

34. Bell, K & Blenkinsop, J. Nd and Sr isotopic composition of East African carbonatites: implications for mantle heterogeneity. *Geology* **15**, 99–102 (1987)

35. Bell, K & Simonetti, A. Carbonatite magmatism and plume activity: Implications from the Nd, Pb and Sr isotope systematics of Oldoinyo Lengai. *J Petrol* **37**, 1321–1339 (1996)

36. Simonetti, A., Bell, K & Viladkar, S.G. Isotopic data from the Amba Dongarcarbonatite complex, west-central India: evidence for an enriched mantle source. *Chem Geol* **122,** 185–198 (1995)

37. Guo, Z.-F. et al. Potassic Magmatism in Western Sichuan and Yunnan Provinces, SE Tibet, China: Petrological and Geochemical Constraints on Petrogenesis. *J Petrol* **46**, 33–78 (2005).

38. Zindler, A. & Hart, S. R. Chemical geodynamics. *Annu Rev Earth Planet Sci* **14**, 493–571 (1986)

39. Hofmann, A.W., 1997. Mantle geochemistry: the message from oceanic volcanism. *Nature* **385**, 219– 229.

40. Bell, K. & Tillton, G.R. Nd, Pb and Sr isotopic compositions of East Africa carbonatites: Mantle mixing and plume inhomogeneity. *J Petrol* **42**, 1927–1945 (2001)

41. Niu, H.C., Shang, Q., Chen, X.M & Zhang, H.X. Relationship between light rare earth deposits and mantle processes in the Panxi rift, China. *Sci China Earth Sci* **46**, 41–49 (2003)

42. Yang, G.M., Chang, C., Zuo, D.H & Liu, X.L. Geology and Mineralization of the Dalucao REE Deposit in Dechang County, Sichuan Province. Open file of China University of Geosciences, Wuhan, pp.1–89 (1998) (in Chinese)

43. Comin-Chiaramonti, P. et al. Mato preto alkaline-carbonatite complex: Geochemistry and isotope (O-C, Sr-Nd) constraints. *Geochim Brazil* **15**, 23–34 (2001)

44. D'Orazio. M., Innocenti, F., Tonarini, S & Doglioni, C.C. Carbonatites in a subduction system: The Pleistocene alvikites from Mt. Vulture (southern Italy). *Lithos* **98** 313–334 (2007)

45. Shivdasan, P.A. Petrology, geochemistry, and mineralogy of pyoxene and pegmatitic carbonatite and the associated fluorspar deposit Okorusu alkaline igneous carbonatite complex, Namibia. Ph.D. dissertation, University of Missouri-Rolla. (2003)

Table S1 Salien characteristics of the CARDs in China

Features	Description
Tectonic setting	Forming along Archean cratonic edges or Proterozoic lithospheric boundaries, during the breakup, destruction, collision-induced reworking of cratons
Host rock	Occurring in clusters along old, reactivated basement rift structures or Cenozoic strike-slip faults, preferentially hosted by carbonatite sill, dyke, stocks, associated with syenite as carbonatite-alkalic complex (e.g., Maoniuping, Dalucao), a few by carbonate strata intruded by carbonatite bodies and ultramafic rocks (e.g., Bayan Ob).
Alteration	Similar hydrothermal alteration: fenitization and K-silicate alteration without alteration zoning
Mineralization	A wide variety of mineralization styles, varying from stockwork or vein system (e.g., Maoniuping) and breccia pipe-hosted (e.g., Dalucao) to disseminated in carbonatite stock (e.g., Lizhuang) and replaced strata-bound ores in carbonates (e.g., Bayan Obo).
Ore paragenesis	Similar paragenesis: bastnaesite being the most significant REE-hosted mineral, and guange assemblage consisting of calcite + barite + fluorite+quartz. Ore signature is LREE-Fe-Nb (e.g., Bayan Obo) and REE-Ba-Sr (e.g., Maoniuping)
Hydrothermal fluids	Similar source and evolution of ore-forming fluids: REE-rich, high-Ba, -K, H_2O-CO_2-SO_4 fluids derived from carbonatitic melt, undergone immiscibility of CO_2- and H_2O-rich fluids with precipitation of bastnaesite at 350-250°C.
Typical deposit and ore ages	Bayan Obo with 1300 Ma overprinted at 380-440 Ma[1-3]; Maoniuping, Dalucao and Lizhuang[4-6] with bastnaesite U-Pb age of 30 Ma, 20.8 Ma and 33 Ma[7], respectively; Laiwu with Rb-Sr age of 118 Ma[8]

Table S2

Sample no.	Rock type	SiO_2	TiO_2	Al_2O_3	TFe_2O_3	Fe_2O_3	FeO
**Dalucao deposit**							
DLC11-9	Carbonatite	20.92	0.37	0.06	0.24		
DLC11-19	Carbonatite	1.91	1.38	0.12	0.57		
DLC11-20	Carbonatite	4.66	2.21	0.16	1.83		
DLC11-21	Carbonatite	1.24	2.64	0.22	0.45		
DLC001-1	Carbonatite	23.50	0.05	0.15	0.99	0.45	0.59
DLC001-3	Carbonatite	17.10	0.02	0.14	1.04	0.58	0.52
DLC038-1	Carbonatite	1.94	0.01	0.11	0.45	0.16	0.31
DLC040-1	Carbonatite	6.87	0.02	0.19	1.57	1.47	0.25
DLC002-1	Syenite	66.40	0.31	16.00	2.12	1.90	0.41
DLC002-2	Syenite	67.40	0.07	14.75	1.72	1.41	0.45
DLC003-1-1	Syenite	68.10	0.16	15.60	2.35	1.83	0.70
DLC003-1-2	Syenite	63.10	0.19	16.80	3.34	2.95	0.68
DLC006-3	Syenite	61.20	0.44	14.20	5.43	5.29	0.66
DLC036-1	Syenite	68.05	0.32	14.95	2.72	1.84	1.06
DLC103-1	Syenite	61.10	0.32	17.00	4.26	3.92	0.74
**Maoniuping deposit**							
MNP11-1-5	Carbonatite	0.52	0.09	0.21	0.36		
MNP-118	Carbonatite	1.86	<0.01	0.23		<0.05	0.45
MNP-125	Carbonatite	7.06	0.08	0.15	1.53	0.70	0.90
MNP-129	Carbonatite	1.33	<0.01	0.09	0.73	0.16	0.59
MNP-131	Carbonatite	2.74	<0.01	0.05	0.90	0.22	0.70
MNP-147	Carbonatite	1.79	<0.01	0.23		<0.05	0.57
MNP09-04-10	Carbonatite	16.30	0.08	1.31	3.40	2.74	0.93
MNP-11	Carbonatite	1.67	0.00	0.09	0.42	0.36	0.10
MNP-16	Carbonatite	2.88	0.00	0.09	0.34	0.20	0.16
MO-48	Syenite	67.52	0.22	14.71		1.61	
MO-52	Syenite	69.15	0.21	14.74		1.51	
MO-62	Syenite	68.94	0.24	14.80		1.32	
MNP-24	Syenite	70.92	0.22	15.97	1.27	0.93	0.43
**Lizhuang deposit**							
LZ11-1-4	Carbonatite	1.27	2.76	0.32	1.26		
LZ11-1-5	Carbonatite	2.32	2.00	0.44	1.49		
LZ11-1-6	Carbonatite	1.26	2.11	0.25	1.13		
LZ11-1-7	Carbonatite	3.75	3.08	0.61	1.35		
LZ11-1-8	Carbonatite	1.08	1.81	0.31	1.21		
LZ09-02-1	Syenite	75.83	0.07	12.99	1.17	1.06	0.22
LZ-07	Syenite	74.75	0.08	13.29	1.00	0.66	0.41

MnO	MgO	CaO	Na_2O	K_2O	P_2O_5	H_2O^+	SrO	BaO
0.10	0.01	23.36	0.10	0.01	0.03		6.46	2.15
0.30	0.01	43.30	0.11	0.01	0.08		1.96	8.25
0.30	0.01	36.45	0.63	0.02	0.45		2.01	9.14
0.14	0.01	36.75	0.12	0.02	0.10		2.12	8.16
0.21	0.05	33.40	0.05	0.03	0.38	1.18	5.97	2.79
0.20	0.03	40.40	0.04	0.08	0.21	0.98	4.74	1.00
0.24	0.02	42.90	0.04	0.09	0.18	0.84	3.24	7.34
0.21	0.03	46.90	0.14	0.06	1.07	0.74	1.18	0.23
0.08	0.16	0.68	3.98	7.77	0.14	0.94	0.30	0.86
0.05	0.10	0.49	2.66	9.48	0.03	0.96	0.09	0.19
0.07	0.16	1.17	5.25	5.85	0.05	1.03	0.24	0.46
0.12	0.06	3.19	4.47	8.60	0.04	0.18	0.44	0.56
0.10	0.19	2.51	2.98	8.19	0.47	1.36	0.08	0.60
0.08	0.70	1.55	5.88	1.74	0.12	1.10	0.06	0.17
0.18	0.17	2.20	4.90	7.54	0.03	0.66	0.58	0.39
0.68	0.01	52.25	0.08	0.03	0.02			
0.95	0.17	48.23	0.15	0.05	0.11	1.10		
0.71	1.95	46.75	0.79	0.25	0.05	1.20		
0.77	0.41	50.04	0.16	0.10	0.05	0.64		
0.79	0.67	50.88	0.36	0.13	0.04	1.14		
0.81	0.32	51.33	0.18	0.15	0.05	1.22		
0.73	1.50	39.60	1.99	0.30	0.05	1.20		
0.60	0.10	55.40	0.06	0.01	0.00	0.70		
0.65	0.10	54.20	0.07	0.12	0.00	0.43		
0.04	0.39	1.86	5.69	5.18	0.12			
0.04	0.45	1.47	5.73	4.71	0.08			
0.03	0.30	1.17	5.08	5.94	0.11			
0.05	0.35	0.15	3.97	5.78	0.05	1.34		
0.51	0.05	26.17	0.13	0.09	0.27			
0.58	0.01	29.69	0.13	0.21	0.30			
0.63	0.14	33.27	0.13	0.09	0.19			
0.39	0.21	21.41	0.18	0.17	0.41			
0.72	0.18	33.46	0.26	0.09	0.14			
0.01	0.11	0.34	5.47	3.51	<0.01	0.60		
0.01	0.16	0.51	5.46	3.63	0.02	0.18		

CO_2	SO_3	LOI	La	Ce	Pr	Nd	Sm	Eu
		9.95	7361.0	9517.0	761.0	2108.0	163.00	39.20
		36.18	865.0	1351.0	125.0	385.0	45.30	12.60
		30.64	2094.0	3607.0	352.0	1081.0	116.00	28.50
		31.55	848.0	1309.0	120.0	372.0	45.20	11.60
24.1	5.46		5269.0	7604.0	739.0	1829.0	159.00	42.70
31.7	2.16		1187.0	1697.0	155.0	400.0	35.50	9.85
32.1	3.02		1198.0	1961.0	206.0	603.0	65.20	16.30
31.4	0.30		10120.0	16510.0	1698.0	4491.0	413.00	99.80
0.25	0.00		93.2	87.0	17.2	52.5	6.33	1.11
0.34	0.00		305.0	359.0	42.3	114.0	10.80	2.49
0.88	0.48		160.0	154.0	21.3	57.9	6.32	1.38
0.25	0.00		254.0	230.0	38.0	113.0	13.40	3.60
0.42	0.00		273.0	260.0	41.4	126.0	16.40	3.96
0.57	0.00		63.3	59.7	10.1	31.4	4.50	0.72
0.43	0.00		238.0	229.0	40.9	125.5	14.80	3.54
		41.59	655.0	1247.0	134.0	478.0	66.30	17.70
38.02	1.73	38.27	2158.0	6371.0	338.0	1653.0	202.00	45.80
25.48	1.58	29.23	1194.0	3632.0	185.0	568.0	106.00	24.00
40.41	1.18	40.46	497.0	1499.0	85.6	557.0	73.90	18.20
40.94	0.18	41.16	220.0	773.0	86.4	324.0	50.70	12.80
40.08	0.45	41.41	260.0	855.0	95.8	354.0	54.80	14.30
26.08	1.28	30.05	420.0	1305.0	103.0	401.0	47.30	11.10
40.10			524.0	1143.0	120.0	468.0	74.30	16.40
40.20			514.0	1079.0	110.0	422.0	63.50	14.40
1.89			381.0	651.0	66.0	183.0	23.00	3.30
1.91			158.0	285.0	32.0	94.0	14.00	3.80
1.24			625.0	1125.0	113.0	300.0	35.00	2.10
0.05			554.0	870.0	102.0	322.0	33.40	7.08
		21.77	1362.0	1815.0	176.0	600.0	72.40	24.80
		24.71	995.0	1414.0	143.0	496.0	61.20	22.40
		28.63	1091.0	1573.0	154.0	515.0	57.90	18.90
		17.79	1927.0	2336.0	212.0	677.0	71.30	22.00
		28.48	1362.0	1809.0	172.0	576.0	67.30	19.80
<0.02	0.09	0.46	89.1	164.0	29.0	94.0	9.70	1.90
0.23			42.6	63.3	5.9	17.5	2.03	0.50

Gd	Tb	Dy	Ho	Er	Tm	Yb	Lu	Totoal REE
49.80	13.60	25.80	3.68	14.70	0.74	4.22	0.44	20062
27.50	4.54	16.10	2.73	9.00	0.95	5.63	0.67	2851
65.60	10.30	28.60	4.41	14.70	1.24	7.59	0.99	7411
28.20	4.44	15.30	2.60	8.07	0.81	4.68	0.55	2770
81.00	10.10	39.20	5.96	17.90	1.70	8.87	1.04	15808
20.50	2.46	10.30	1.71	5.11	0.58	3.49	0.43	3529
36.50	3.97	17.10	2.66	7.64	0.81	4.41	0.53	4123
205.00	25.10	103.00	16.10	46.90	4.72	23.50	2.52	33759
3.67	0.51	2.28	0.41	1.11	0.15	0.95	0.13	267
6.27	0.66	2.77	0.44	1.28	0.14	0.81	0.11	846
4.01	0.51	2.34	0.39	1.29	0.18	1.30	0.18	411
8.72	1.00	4.77	0.82	2.26	0.29	1.88	0.28	672
10.50	1.28	6.17	1.04	2.89	0.38	2.30	0.30	746
3.97	0.59	3.55	0.70	1.85	0.28	1.60	0.22	182
9.39	1.10	5.26	0.89	2.54	0.37	2.17	0.33	674
47.20	6.93	27.50	4.82	15.50	1.70	10.90	1.46	2714
170.00	17.00	65.90	11.90	37.1	4.24	26.90	3.41	11104
88.50	8.06	28.10	5.07	16.4	1.88	12.30	1.67	5871
63.90	6.68	28.00	5.09	15.2	1.85	12.00	1.61	2856
42.60	5.25	24.10	4.45	13.3	1.66	10.90	1.51	1571
45.50	5.58	25.20	4.75	14.3	1.77	11.80	1.59	1744
39.60	3.96	15.80	2.81	8.68	1.04	7.32	1.07	2368
53.00	6.27	29.70	5.04	15.10	1.94	12.00	1.53	2470
45.50	5.20	24.40	4.33	13.10	1.65	10.80	1.33	2309
14.00	1.10	4.40	0.70	1.70	0.20	1.60	0.20	1331
9.30	1.10	4.20	0.60	1.80	0.30	1.40	0.20	606
21.00	1.90	5.70	0.80	2.00	0.30	1.70	0.20	2234
20.50	1.85	7.10	1.18	3.76	0.40	2.54	0.38	1926
49.60	6.43	19.90	3.39	11.30	1.20	7.78	1.14	4151
41.30	5.66	18.00	3.02	11.00	1.11	7.71	1.13	3221
37.40	5.25	15.30	2.68	9.61	0.98	6.51	0.96	3488
41.20	6.25	16.60	2.80	9.96	0.95	6.30	0.89	5330
42.20	6.27	20.00	3.54	11.90	1.26	8.50	1.22	4101
5.40	0.50	1.50	0.25	0.95	0.12	0.96	0.17	144
1.33	0.16	0.68	0.13	0.46	0.07	0.51	0.08	135

Y	Rb	Sr	Ba	Th	U	Pb	Nb	Ta	Zr	Hf
47	7.1	54590	19220	36.2	38.1	640	0.5	0.05	0.7	0.33
83	4.1	16570	73890	4.9	16.0	736	33.6	0.06	4.2	0.34
104	4.5	17030	81880	24.8	74.6	1137	127.0	0.11	37.3	0.94
69	5.0	17950	73070	3.0	25.8	1354	57.6	0.10	5.3	0.35
173	1.4	50520	24960	18.1	50.6	510	16.3	0.31	18.9	0.90
60	1.9	40100	8970	5.2	5.0	487	3.1	0.23	8.5	0.36
87	10.4	27380	65780	9.2	18.6	568	40.6	0.18	5.3	0.19
544	3.4	10020	2031	139.0	131.0	1514	2.3	0.22	23.5	1.11
11	187.0	2571	7663	25.1	4.9	146	26.4	0.81	322.0	8.02
14	296.0	780	1719	118.0	10.9	103	25.0	0.72	577.0	18.00
14	139.0	2001	4094	56.6	11.5	136	32.6	0.82	758.0	16.80
32	189.0	3752	4999	61.1	13.1	180	33.0	0.99	771.0	19.10
36	251.0	694	5354	34.2	7.4	1714	16.3	0.75	366.0	8.69
16	29.2	483	1541	6.1	0.9	44	12.1	0.37	97.0	2.44
31	164.0	4920	3516	76.7	12.4	175	43.5	1.14	1402.0	28.90
144	5.1	8706	5864	1.1	0.5	451	0.9	0.08	0.4	0.32
232	2.5	8302	25900	104.0	33.2	1707	4.7	0.09	1.0	0.26
159	4.0	7987	27870	61.2	37.0	756	88.0	0.70	12.8	1.13
134	5.8	10230	20580	6.7	7.1	778	2.9	<0.05	1.6	0.16
124	4.2	10010	1071	2.3	0.6	141	1.3	<0.05	2.0	0.21
131	5.3	9843	7547	2.0	0.9	223	0.8	<0.05	4.5	0.27
84	20.0	8846	22630	61.2	46.7	504	63.0	0.44	35.9	1.81
149	0.3	10781	1629	0.7	0.1	102	0.3	0.02	0.1	0.05
133	1.2	11677	1295	1.0	0.2	104	0.0	0.01	0.1	0.05
22	194.0	1885	2418	57.0	20.0	135	31.0	1.00	192.0	4.80
22	197.0	728	2145	53.0	9.9	96	21.0	0.90	194.0	4.10
24	214.0	2004	3044	50.0	18.0	91	48.0	1.30	201.0	5.10
37	163.0	225	1384	60.0	5.0	930	14.8	0.72	201.0	6.21
44	10.3	30480	20740	63.7	15.9	210	6.6	0.14	1.4	0.39
46	13.4	29990	28910	35.8	7.3	117	1.3	0.09	0.5	0.34
39	10.1	27430	35100	24.3	3.9	152	6.9	0.12	1.2	0.35
33	13.9	31060	14730	12.7	2.1	170	0.7	0.05	0.3	0.26
49	11.7	28390	34740	9.8	2.1	136	1.0	0.04	0.1	0.25
7	114.0	146	1436	101.0	35.6	23	45.8	1.26	319.0	14.10
5	135.0	181	186	47.2	6.1	44	13.0	0.63	81.2	4.20

Sc	References
0.09	This study
4.54	This study
5.70	This study
3.11	This study
3.74	This study
3.41	This study
3.07	This study
4.26	This study
1.68	This study
1.46	This study
1.64	This study
1.49	This study
4.53	This study
2.45	This study
2.44	This study
2.55	This study
1.21	This study
3.38	This study
1.07	This study
1.70	This study
0.84	This study
4.15	This study
2.45	Xu et al., 2002
1.91	Xu et al., 2002
	Hou et al., 2006
	Hou et al., 2006
	Hou et al., 2006
1.36	Hou et al., 2006
0.77	This study
1.64	This study
2.47	This study
0.59	This study
1.38	This study
2.70	This study
0.16	Hou et al., 2006

Table S3

Sample no.	Mineral and rock	$^{206}Pb/^{204}Pb$	$^{207}Pb/^{204}Pb$	$^{208}Pb/^{204}Pb$	$^{87}Rb/^{86}Sr$	$^{87}Sr/^{86}Sr$	$(^{87}Sr/^{86}Sr)_i$	$^{147}Sm/^{144}Nd$	$^{143}Nd/^{144}Nd$	$\varepsilon_{Nd(t)}$	T_{DM}(Ga)	References
**Dalucao deposit**												
DLC11-9	Carbonatite	18.2050	15.6254	38.613	0.000388	0.707527	0.707527	0.04674	0.512358	-5.2	0.72	This study
DLC11-19	Carbonatite	18.2098	15.6266	38.626	0.000741	0.707596	0.707596	0.07113	0.512348	-5.5	0.86	This study
DLC11-20	Carbonatite	18.2219	15.6287	38.638	0.000793	0.707696	0.707696	0.06487	0.512346	-5.5	0.82	This study
DLC11-21	Carbonatite	18.2135	15.6285	38.635	0.000825	0.707266	0.707266	0.07345	0.512347	-5.5	0.87	This study
DLC001-1	Calcite in carb.	18.2720	15.7090	38.897	0.001800	0.707848	0.707848					This study
DLC001-3	Calcite in carb.	18.2700	15.7080	39.066	0.000400	0.707863	0.707863	0.06090	0.512316	-6.0	0.83	This study
DLC001-5	Calcite in carb.	18.2230	15.6520	38.705	0.000200	0.707863	0.707863	0.04570	0.512327	-5.8	0.75	This study
DLC038-1	Calcite in carb.	18.2700	15.7010	38.888	0.000100	0.707962	0.707962	0.07070	0.512325	-5.9	0.88	This study
DLC040-1	Calcite in carb.	18.2700	15.7130	38.919	0.000600	0.70779	0.707790	0.06200	0.512341	-5.6	0.82	This study
**Maoniuping deposit**												
MNP11-1-5	Carbonatite	18.2236	15.6080	38.505	0.001729	0.705897	0.705896	0.08385	0.512439	-3.6	0.84	This study
MNP-118	Carbonatite	18.2157	15.5947	38.455	0.000900	0.706146	0.706146	0.07430	0.512463	-2.7	0.75	This study
MNP-125	Carbonatite	18.2310	15.5929	38.451	0.001500	0.706147	0.706146	0.11340	0.512487	-2.4	1.01	This study
MNP-129	Carbonatite	18.2202	15.5952	38.456	0.001700	0.70617	0.706169	0.08070	0.512449	-3.0	0.81	This study
MNP-131	Carbonatite	18.2236	15.5938	38.460	0.001200	0.706182	0.706181	0.09510	0.512445	-3.2	0.91	This study
MNP-147	Carbonatite	18.2206	15.5935	38.453	0.001600	0.706156	0.706155	0.09410	0.512417	-3.7	0.94	This study
MNP09-04-10	Carbonatite	18.2243	15.5940	38.457	0.006600	0.706129	0.706126	0.07170	0.512518	-1.6	0.68	This study
MN-1-2	Carbonatite	18.1740	15.5450	38.301	0.000163	0.706320	0.706320	0.10060	0.512357	− 5.1	1.07	Wang et al., 2001
MN-1-4	Carbonatite	18.1720	15.5360	38.283	0.001510	0.706050	0.706050	0.10810	0.512436	− 3.5	1.03	Wang et al., 2001
MN-2-7	Carbonatite	18.1620	15.5410	38.295	0.000139	0.706160	0.706160	0.08880	0.512327	− 5.6	1.01	Wang et al., 2001
MN-2-10	Carbonatite	18.1770	15.5470	38.306	0.000106	0.706170	0.706170	0.08860	0.512396	− 4.2	0.92	Wang et al., 2001
MN-2-6	Carbonatite	18.1870	15.5670	38.390	0.000468	0.706911	0.706911	0.09630	0.512408	− 4.0	0.97	Wang et al., 2001
MN-40	Carbonatite	18.1940	15.5510	38.352	0.000309	0.706280	0.706280	0.10440	0.512403	− 4.2	1.04	Wang et al., 2001
MO-67	Calcite in carb.					0.706240	0.706240	0.07946	0.512413	− 3.9	0.84	Hou et al., 2006
MO-69	Calcite in carb.					0.706330	0.706330	0.04904	0.512407	− 3.8	0.69	Xu et al., 2002
MNP-13	Calcite in carb.	18.2230	15.5380	38.323	0.000855	0.706020	0.706020	0.08363	0.512405	− 4.0	0.88	Xu et al., 2002
MNP-16	Calcite in carb.	18.2640	15.5560	38.683	0.000054	0.706075	0.706075	0.10010	0.512405	− 4.1	1.00	Xu et al., 2002
MNP-125	Calcite in carb.	18.2200	15.5870	38.452	0.000052	0.706149	0.706149	0.09825	0.512411	− 4.0	0.98	Xu et al., 2002
MNP-135	Calcite in carb.				0.000073	0.706074	0.706074	0.09456	0.512411	− 4.0	0.95	Xu et al., 2002
**Lizhuang deposit**												
LZ-127	Carbonatite	18.1926	15.5873	38.357	0.000600	0.706195	0.706195	0.05150	0.512506	-1.8	0.61	This study
LZ11-1-4	Carbonatite	18.2126	15.6043	38.426	0.001007	0.705674	0.705674	0.07295	0.512453	-3.2	0.76	This study
LZ11-1-5	Carbonatite	18.2090	15.6006	38.416	0.001332	0.705733	0.705732	0.07459	0.512449	-3.1	0.77	This study
LZ11-1-6	Carbonatite	18.2109	15.6033	38.418	0.001098	0.705624	0.705624	0.06797	0.512452	-3.0	0.73	This study
LZ11-1-7	Carbonatite	18.2172	15.6036	38.425	0.001334	0.705734	0.705733	0.06367	0.512455	-3.0	0.71	This study
LZ11-1-8	Carbonatite	18.2113	15.6045	38.423	0.001229	0.705789	0.705789	0.07063	0.51246	-2.9	0.74	This study
LZ-01	Calcite in carb.	18.2201	15.6017	38.434	0.004401	0.706305	0.706305	0.12314	0.512372	-4.9	1.31	Hou et al., 2006
LZ-03	Calcite in carb.	18.1965	15.6014	38.401	0.003050	0.706713	0.706713	0.15472	0.512412	-4.2	1.91	Hou et al., 2006
LZ-09	Calcite in Carb.	18.2010	15.6025	38.408	0.002051	0.706997	0.706997	0.09659	0.512432	-3.6	0.94	Hou et al., 2006
LZ-17	Calcite in Carb.	18.2069	15.6038	38.424	0.001249	0.706314	0.706314	0.05653	0.512441	-3.2	0.69	Hou et al., 2006

Table S4

Sample no.	Mineral and roc	$\delta^{13}C_{V-PDB}$	$d^{18}O_{V-SMOW}$	References
**Dalucao deposit**				
DLC11-7	Carbonatite	-7.5	8.8	This study
DLC11-9	Carbonatite	-6.1	9.8	This study
DLC11-17	Carbonatite	-6.6	8.3	This study
DLC11-19	Carbonatite	-8.0	8.7	This study
DLC11-20	Carbonatite	-8.2	8.0	This study
DLC11-21	Carbonatite	-7.9	8.9	This study
DLC137	Carbonatite	-8.0	8.8	This study
DLC001-1	Calcite in carb.	-8.2	8.4	This study
DLC001-3	Calcite in carb.	-8.2	8.5	This study
DLC001-5	Calcite in carb.	-8.0	8.5	This study
DLC038-1	Calcite in carb.	-8.7	8.2	This study
DLC040-1	Calcite in carb.	-8.8	8.2	This study
DL9614	Carbonatite	-8.3	7.7	Yang et al.,1998
DL9667(1)	Carbonatite	-5.9	8.6	Yang et al.,1998
DL9667(2)	Carbonatite	-7.4	7.6	Yang et al.,1998
DL9614B	Calcite in carb.	-8.5	7.5	Yang et al.,1998
DL9667R	Calcite in carb.	-7.2	6.7	Yang et al.,1998
DL9667H	Calcite in carb.	-7.4	7.4	Yang et al.,1998
**Maoniuping deposit**				
MNP11-1-5	Carbonatite	-6.5	8.3	This study
MNP11-1-16	Carbonatite	-6.7	7.9	This study
MNP11-1-3	Carbonatite	-6.8	8.4	This study
MNP11-1-6	Carbonatite	-6.6	8.2	This study
MNP11-1-7	Carbonatite	-6.7	8.2	This study
MNP11-1-11	Carbonatite	-6.6	8.2	This study
MNP-118	Calcite in carb.	-6.7	7.9	This study
MNP-125	Calcite in carb.	-6.5	7.6	This study
MNP-129	Calcite in carb.	-6.6	7.4	This study
MNP-131	Calcite in carb.	-6.5	7.5	This study
MNP-146	Calcite in carb.	-6.5	7.7	This study
MNP-147	Calcite in carb.	-6.6	7.5	This study
MO-67	Calcite in carb.	-6.6	7.7	Hou et al., 2006
MO-68	Calcite in carb.	-6.3	7.8	Hou et al., 2006
MO-69	Calcite in carb.	-6.3	7.7	Hou et al., 2006
MNP-6	Calcite in carb.	-6.8	7.2	Xu et al., 2002
MNP-11	Calcite in carb.	-6.6	6.4	Xu et al., 2002
MNP-13	Calcite in carb.	-6.7	7.2	Xu et al., 2002
MNP-15-1	Calcite in carb.	-7.0	6.7	Xu et al., 2002
MNP-16-1	Calcite in carb.	-6.7	7.2	Xu et al., 2002
MNP-125	Calcite in carb.	-6.8	6.9	Xu et al., 2002
3-6H	Calcite in carb.	-6.6	6.4	Niu et al., 2005
M014	Calcite in carb.	-6.0	7.4	Niu et al., 2005
M015	Calcite in carb.	-6.1	7.5	Niu et al., 2005
M016	Calcite in carb.	-6.6	7.5	Niu et al., 2005

M014-1	Calcite in carb.	-6.7	7.5	Niu et al., 2005
M015-1	Calcite in carb.	-6.6	7.5	Niu et al., 2005
**Lizhuang deposit**				
LZ-127	Carbonatite	-4.5	8.8	This study
LZ11-1-4	Carbonatite	-6.5	8.3	This study
LZ11-1-5	Carbonatite	-6.5	8.5	This study
LZ11-1-6	Carbonatite	-6.4	8.5	This study
LZ11-1-7	Carbonatite	-6.3	8.1	This study
LZ11-1-8	Carbonatite	-6.3	8.7	This study
LZ11-1-11	Carbonatite	-6.5	8.6	This study
LZ-03	Calcite in carb.	-4.6	9.6	Hou et al., 2006
LZ-09	Calcite in carb.	-4.4	9.0	Hou et al., 2006
LZ-17	Calcite in carb.	-4.7	8.7	Hou et al., 2006

Table S5

Samples	Rock type	SiO_2	TiO_2	Al_2O_3	TFe_2O_3	Fe_2O_3	FeO	MnO	MgO	CaO	Na_2O	K_2O	P_2O_5	H_2O^+	CO_2	S	LOI	Total	La	Ce	Pr	Nd	Sm	Eu	Gd	Tb
**Bayan Obo**																										
BN09-13	carbonatite	1.61	0.02	0.14		0.26	1.33	0.36	8.82	15.61	0.26	0.07	7.6			1.03	25.68		69400	90450	6846	17130	1526	338	1195	74.6
BN09-35	carbonatite	6.59	0.12	0.98		5.95	2.29	1.46	8.45	30.56	0.13	0.73	3.99			0.01			4872	7851	730	2561	401	98.8	240	24
BN09-48	carbonatite	0.42	0.01	0.05		0.06	1.49	0.37	17.23	33.36	0.11	0.02	3.61						445	559	89.7	328	49.2	13.1	37.9	4.15
M-3-1	calcite carbonatite	2.3	0	0.3	0.54		0.44	0.55	1.23	46.51	0.38	0.05	0.09				41.08	93.03	1409	2455	269	776	85.9	18.5	48.5	5.36
05B123	calcite carbonatite	12.43	0.29	1.53	5.62		2.52	0.35	8.34	36.22	0.10	2.27	1.29				25.03	93.47	955	2411	308	1063	122	27.5	80.2	11.3
M-12-1	calcite carbonatite	7.22	0.02	0.27	0.86		0.34	0.14	0.17	28.26	0.00	0.24	0.06				32.05	69.29	55937	95227	10362	27425	3255	669	1557	125
05B006	calcite carbonatite	3.45	0	0.06	0.41		0.34	0.33	0.25	25.77	0.05	0.07	0.33				29.49	60.21	54622	70300	5840	12243	1004	193	589	46.3
BY177	calcite carbonatite																		5283	6956	511	1600	107	25	54.1	17.2
BY180	calcite carbonatite																		53080	72990	5843	15998	1230	240	498	80
BY181	calcite carbonatite																		43185	52001	3465	9445	801	176	421	84
BY184	calcite carbonatite																		94810	90630	5392	6538	492	49	458	52
**Laiwu deposit**																										
0922XY-1	ferro carbonatite	13.56	0.62	1.93		9.02		0.29	5.34	31.59	0.02	1.65	4.87				29.69	98.58	829	1660	210	801	104	24.4	70.6	7.7
0922XY-2	ferro carbonatite	14.2	0.76	2.82		9.61		0.23	5.46	30.83	0.08	1.79	4.88				26.57	97.23	745	1503	193	730	94.9	21.6	65.4	6.84
HJZ-4-1	calcio carbonatite	2.93	<0.01	0.27		4.53		0.53	0.57	46.93	0.01	0.21	2.62				36.06	94.58	1350	2607	287	1039	138	32.4	97.2	9.68
HJZ-4-2	calcio carbonatite	3.65	<0.01	0.23		5.94		0.49	0.61	46.15	<0.01	0.14	3.46				34.69	95.28	1265	2549	305	1027	161	37.6	107	11.1
0922BD-1	ferro carbonatite	30.14	1.02	6.84		8.31		0.12	8.2	19.63	1.9	3.76	2.81				16.93	99.66	233	474	65.4	264	38.5	8.79	25.9	2.81
0922BD-2	ferro carbonatite	36.05	0.61	9.63		6.16		0.21	4.65	20.94	4.02	1.69	1.27				14.36	99.59	236	549	76.1	315	44.5	10.2	29.9	3.27
YJQ-2	calcio carbonatite	11.08	0.18	1.49		2.88		0.33	4.36	39.61	0.04	0.31	4.82				31.9	97	1026	2008	212	787	99.6	23.3	70.9	6.36
YMQ-1	calcio carbonatite	14.81	0.18	1.03		2.43		0.25	1.38	42.7	0	0.23	4.32				30.99	98.32	1021	2018	180	780	96.8	23	70.3	6.27
YMQ-2	calcio carbonatite	17.77	0.21	1.27		2.6		0.16	1.02	41.3	0.07	0.33	4.37				29.45	98.55	968	1932	197	743	94.5	21.8	68.5	6.09
02LT-1	ferro carbonatite	17.99	0.16	3.55		11.53		0.42	5.33	23.07	0.12	2.75	2.55				26.87	94.34	1149	2361	220	870	113	26.5	80.8	7.32
02LT-2	magnesio carbonatite	20.69	0.74	5.45		8.22		0.2	9.46	21.86	0.21	3.12	3.86				20.82	94.63	529	1225	143	552	78.6	18.8	56.2	5.38
DSM-1	ferro carbonatite	9.02	0.08	2.82		6.68		0.59	2.82	37.58	0.06	0.55	3.11				28.87	92.18	2334	4462	546	2048	250	55.3	156	14.8
02DSM-1	magnesio carbonatite	7.39	0.13	1.37		6.9		0.3	8.53	32.35	0.03	0.35	6.27				32.38	96	782	1653	190	712	92.8	21.6	64.7	5.6
**Mountain Pass deposit**																										
85--4	bastnaesite-barite soevite	0.4	0.01	0.01		0.24		0.24	0.04	21.3	0.05	0.05	0.04			7.30		99.83		37931						
Q-924	bastnaesite-barite beforsite	1.63	0.01	0.01		1.77		0.45	6.41	11.73	0.07	0.09	0.29			6.17		98.20		50112						
Q-822	bastnaesite-barite beforsite	2.2		0.17		1.8		0.46	4.7	10.4			0.18			6.8		95.74								
Q-824	bastnaesite-barite beforsite	7.7		0.14		2.7		0.54	5.7	17.3			0.39			2.8		94.23								
R-757	bastnaesite-barite beforsite	0.5		0.02		6.6		1.4	14.6	26.8			0.34			0.32		99.95								
Q-833	bastnaesite-barite ddomitic soevite	14.1		0.08		2.4		0.51	2.4	22.2			0.34			1.64		96.15								
R-711	bastnaesite-barite ddomitic soevite	1.3		0.02		1.6		0.38	1.2	12.7			0.57			4.6		93.92								
R-744	bastnaesite-barite ddomitic soevite	1.5		0.04		4.3		0.83	5.3	21.4			0.58			3.32		96.82								
R-733	bastnaesite-barite ddpmitic soevite di	1.4		0.03		2.2		0.52	8.1	20.1			1.70			4.64		99.07								
**Southern Brazil deposit**																										
2/16.20	carbonatite	0.69	<0.01	0.01		0.82	8.23	2.17	14.38	29.04	0.04	0.01	0.02	0.02	44.81	0.04		100.33	52.5	97.8	10.7	36.5	6.9	2.49	7.33	1.02
2/31.00	carbonatite	1.02	<0.01	0.05		1.02	5.75	1.61	15.81	29.15	0.02	0.02	0.02	0.01	45.28	0.01		100.13	67.8	126	13.5	44.2	8.55	3.25	11	1.65
5/13.00	carbonatite	0.49	0.03	0.01		0.56	5.96	1.65	16.15	29.12	0.01	0.02	0.02	–	45.25	0.01		99.45	68.1	127	13.9	43.8	7.24	1.87	4.53	0.64
4/10.30	carbonatite	0.69	0.03	0.02		1.47	14.01	3.95	9.30	28.85	0.05	0.02	0.02	0.01	43.20	0.10		101.79	90.9	184	22.6	81	12.8	3.09	6.5	0.67
1/34.90	carbonatite	0.52	<0.01	0.03		1.10	8.51	2.96	13.45	29.15	0.09	0.03	0.04	0.01	44.70	0.02		100.69	203	303	29.4	83.2	9.4	2.45	5.86	0.69
**Amba Dongar India F deposit**																										
1	carbonatite	6.5	0.17	0.55		1.2	0.32	0.89	4.23	48.16			0.39		36.52			100.62	243	491.4		143.29	21.93	5.06	12.98	
2	carbonatite	3.3	0.33	0.76		1.75	1.24		2.18	48.27			0.09		40.01			99.95	302	465.34		145.08	21.53	4.75	13.57	
3	carbonatite	3.12	0.11	0.51		1.35	0.36	0.1	1.4	50.11			0.68		39.77			99.84	522	632.6		140.83	19.96	4.35	14.28	
4	carbonatite	6.25	0.64	0.55		1.28	1.32	0.07	3.81	48.23					36.81			100.05	1148	1758		426.85	52.31	10.55	34.22	
5	carbonatite	3.25		0.16		0.93	0.32	0.09	2.08	50.46			0.09		39.69			99.68	1091	1953		733.35	107.31	23.81	61.37	
6	carbonatite	32.38	0.77	14.5		7.17	0.96	0.72	2.41	19.24	0.75	6.57	0.27		11.29			99.24	1293	1868		503.3	67.43	12.04	46.04	
**Northwestn Namibia deposit**																										
OS-4	carbonatite	7.28	0.22	0.21		6.94	0.85	1.28	1.7	40.67	0	0	7.08	1.52	27.04			95.52	2793	4015	372	1141	183	55.3	156	25.4
OS-5	carbonatite	32.18	0.54	8.34		9.64	0	1.55	0.77	18.11	0	7.15	0.06	1.32	15.85			97.03	765	1054	82.2	277	42.7	9.96	20.8	0
K-2	carbonatite	15.85	0.99	4.83		1.54	9.62	1.91	5.02	23.16	1.23	3.24	1.04	1.34	22.13			92.24	4771	7284	519	1566	184	45.3	98	12.6
K-22	carbonatite	1.79	0.18	1.39		20.64	0	4.42	4.84	24.54	0	0	0.44	2.37	27.31			89.94	7232	9835	826	2424	391	95.1	161	13.2
ON-4	carbonatite	9.57	0.03	0.24		16.74	16.24	5.41	5.73	10.57	1.01	0.01	1.87	1.1	23.18			93.32	3771	5858	468	1074	97.2	16.3	34.8	3.16
ON-8	carbonatite	2.94	0.04	0.59		4.67	4.75	3.63	8.09	28.2	0	0.03	4.78	1.61	30.76			90.8	3944	6049	597	1788	132	60.9	132	14.3
ON-9	carbonatite	1.72	0.02	0.23		1.95	5.53	2.16	4.96	35.81	0	0	7.37	0.43	34.68			95.43	1760	3894	487	1533	211	51.7	112	14.2

ON-10	carbonatite	4.68	0.03	0		1.3	5.16	1.21	2.06	42.29	0	0.01	4.36	0.57	33.01			96.03	1225	2296	291	1007	161	46.2	98.9	12
OKU-6	carbonatite	16.4	1.49	3.87		14.43	2.53	0.44	2.62	26.41	0.38	2.12	2.66	0.71	23.1			97.61	1013	1739	152	368	36.6	8.61	13.6	1.64
OKU-18	carbonatite	1.22	0.01	0.49		3.73	0.44	0.58	0.68	47.53	0	0.04	1.34	1.21	36.36			94.12	1488	2857	202	614	67.8	21.2	53	7.39
**Malawi deposit and occurrence**																										
G 357	calcio carbonatite	2.46	0.03	0.07		5.89		0.26	0.37	53.73	0	0.01	1.21		40.22	0.01		104.3								
G369	calcio carbonatite	1.16	0.01	0.09		3.49		0.53	0.47	52.05	0	0.04	1.46		40.18	0.00		99.5								
N 1290	calcio carbonatite	3.68	0.02	0.14		5.64		1	0.71	48.24	0	0	1.30		38.07	0.02		98.8								
N 1295	calcio carbonatite	0.32	0.01	0.04		5.28		0.77	1.77	48.94	0	0	0.85		39.62	0.08		97.7								
G410	pyrochlore-rich carbonatite	28.16	0.32	0.39		6.23		0.55	9.18	23.54	2.14	1.01	4.76		19.48	0.00		95.8								
G413	ankeritic carbonatite	0.17	0.02	0.03		2.64		0.70	12.68	39.23	0	0	0.55		44.35	0.00		100.4								
MW 166	ddomitic carbonatite	6.04	0.02	0.16		4.91		1.22	16.40	26.34	0	0	0.41		41.41	0.02		96.9								
MW168	sideritic carbonatite	5.17	0.03	0.65		52.77		11.49	5.75	8.84	0	0	0.47		17.83	0.43		103.4								
**West Greenland deposit and occurrence**																										
1	carbonatite	0.52	0.01	0.02		0.39	3.66	0.3	14.35	27.53	0.03	0.02	2.3						1200	1680				236		43.8
2	carbonatite	3.27	0.22	0.31		5.06	6.42	0.62	16.78	30.24	0.57	0.15	5.1						1860	3300				200		18.4
3	carbonatite	2.05	0.08	0.13		1.86	4.68	0.41	15.53	28.97	0.26	0.08	3.59						3400	8690		4380		31.2		
4	carbonatite	5.08	0.11	0.78		0.85	3.06	0.22	8.24	14.96	0.03	0.59	0.07						2870	7210				24.4		
5	carbonatite	31.07	0.6	7.23		5.07	7.87	0.49	15.54	26.86	1.54	5.11	1.74						212	631		279	37.9	9.38		1.05
6	carbonatite	17.43	0.31	3.18		2.53	4.7	0.36	11.92	20.81	0.76	2.35	0.86						180	499		341	30.9	6.56		
7	carbonatite	12.1	0.8	3.55		3.12	3.78	0.61	5.64	35.12	0.42	1.49	2.06						346	684		303	94.50	17.20		
8	carbonatite	2.22	0.15	2.01		1.99	6.23	0.9	9.4	30.24	0.26	0.31	1						398	858			43.10	8.66		
**Khibina Kola deposit**																										
632B/1934	carbonatite	4.81	0.64	0.88			4.99	0.37	1.2	46.5	0.36	1.45	1.25			0.32		64.79	437	955		403				
632B/1960	carbonatite	3.68	0.22	0.49			3.02	0.39	0.97	45.95	0.59	0.38	1.67			0.2		59.57	492	1078		418				
633/477.7	carbonatite	0.64	0.03	0.25			3.79	1.56	0.41	32.52	3.09	0.09	0.02			0.73		61.02	28384	37990		7969				
603/165.5	carbonatite	0.19	0.02	0.06			2.29	0.85	0.35	35.12	1.68	0.05	0.04			0.53		62.92	35565	50727		10620				
604/454	carbonatite	0.44	0.01	0.1			5.64	5.86	2.05	28.7	0.14	0.12	0.09			0.74		58.21	18589	31341		6395				
603/225	carbonatite	0.87	0.04	0.29			8.09	11.39	3.72	24.59	0.22	0.03	0.08			0.41		62.19	22891	37558		8852				
603/89	carbonatite	1.88	0.02	0.69			19.14	12.78	3.97	12.13	0.21	0.55	0.11			1.59		58.16	2540	6823		2814				
604/90	carbonatite	2.09	0.08	0.7			22.97	12.6	2.35	18.4	0.25	0.69	0.91			1.12		65.16	4064	8868		3099				
**Okorusu Namibia deposit and occurrence**																										
V	carbonatite	12.2	0.6	0.59		19.8		0.31	1.52	34.8	0.34	0.45	18.7						959	2370	202	721	76.4	18.7	44.5	3.5
92300	pyroxene carbonatite	6.65	1.55	0.64		30.5		0.5	1.13	27.1	0.16	0.43	16.3						1560	3930	319	1160	120	28.7	68.1	5.8
92627	carbonatite	11.6	0.13	0.36		4.62		0.25	2.62	44.1	0.49	0.33	19.2						1480	3630	292	1040	107	25.3	59.9	5.2
92629	carbonatite	6.33	1.05	0.43		18.1		0.48	0.66	34.8	0.03	0.35	4.58						641	1400	120	449	42.7	9.8	22	2
92500	carbonatite	0.86	0.01	0.15		1.06		0.36	0.07	47.6	0.01	0.01	0.78						881	2060	172	575	61.5	17.5	43.9	4.7
92016	carbonatite	19.9	0.88	0.7		6.92		0.44	4.42	37.6	0.35	0.39	5.06						537	1450	107	383	41.6	10.4	25.5	2.5
92012	calcite in carbonatite	2.48	0.01	0.01		0.27		0.1	0.07	49.6	0.05	0.02	4.12						484	1010	83.3	290	30	7.3	17.9	1.8
92519	carbonatite	11.6	0.02	0.06		1.07		0.17	0.17	47.8	0.07	0.01	19.5						1450	3450	277	1010	103	26	29.7	3.9
**Kovdor Kola**																										
Kv12	calcio carbonatite	0.96	0.07	0.38		1.96	1.26	0.07	1.61	51.7		0.2	0.33		41.89			100.43	100	183	20.5	72	10.2	2.89	7.57	
Kv13	calcio carbonatite	1.96	0.03	0.14		1.38	1.08	0.08	3.01	51.57	0.02	0.08	0.89		40.12			100.36	102	190	21.6	76	10.7	3	7.92	
Kv14	calcio carbonatite	3.26	0.1	0.36		6.89	2.31	0.12	5.35	45.03	0.03	0.08	0.11		36.77		37.49	100.4	61	114	12.9	45.7	6.53	1.9	5.11	
Kv15	calcio carbonatite	0.95	0.11	0.19		3.99		0.07	1.87	49.7	0.02	0.18	1.9					96.47	190	467	39.7	140	18.5	4.97	12.6	
Kv16	dolomite carbonatite	0.42	0.03	0.07		1.05	1.53	0.19	19.81	31.05	0	0.05	1.53		44.67			100.4	26.1	56	6.3	22.6	3.2	0.91	2.48	
Kv17	dolomite carbonatite	1.09		0.31		0.92		0.18	20.62	30.96	0.04	0.19	1.08		45.13			100.52	26.8	56	6.42	22.9	3.05	0.83	2.11	
**Mato Preto**																										
Ⅰ.-119.3	carbonatite	0.3	0.01	0.23			1.81	0.19	0.99	54.35	0.03	0.1	0.52				41.27		198.1	377	44.3	155.8	25.8	8.23	25.04	3.74
Ⅰ.-84.0	carbonatite	0.23	0.01	0.14			0.92	0.19	0.33	54.83	0.01	0.07	0.17				43.15		164.6	461.4	69.51	340.8	73.37	22.63	76.23	12.01
Ⅱ.-77.0	carbonatite	0.52	0.11	0.21			2.23	0.22	1.32	53.13	0.03	0.09	0.51				41.38		240.5	716.9	101.01	461.3	91.35	22.2	70.56	8.27
Ⅲ.-70.0	carbonatite	1.91	0.02	0.21			1.73	0.21	0.51	55.01	0.01	0.31	0.16				40.16		3686	6510	581.12	883.7	174.3	56.79	161.39	23.12
Ⅲ.-62.2	carbonatite	2.28	0.11	0.59			10.01	0.73	6.12	39.21	0.1	0.58	0.28				38.89		852	1577	154.6	350.1	52.31	17	48.33	6.91
Ⅲ.0.6227	carbonatite	3.22	0.06	0.81			4.56	0.39	1.49	49.74	0.06	1.09	1.67				36.4		1112	1808	232.9	948.1	79.04	24.99	87.59	14.44
**Uganda, Namibia**																										
UG6	carbonatite lava	11.03	1.62	2.69		7.52	4.14	0.45	8.24	34.86	0.37	0.26	3.17	3.43	19.3	1220		97.08	454.5	896.5		354	46	13.1	30.2	3.48
UG15	carbonatite lava	18.45	2.4	4.9		3.85	6.98	0.35	8.95	28.31	0.45	0.63	2.34	4.27	15.9	530		97.78	318.5	606.1		257.6	33.5	8.57	22	2.41
UG16	carbonatite tuff	15.89	2.02	4.53		6.47	3.33	0.29	4.56	33.29	0.5	0.86	1.94	3.08	22	280		98.76	287.3	526.7		228.9	29.6	7.89	20.8	2.17
UG18	carbonatite tuff	15.82	1.73	4.25		1.26	7.05	0.25	4.44	34.27	0.53	0.94	1.78	2.53	23.6	360		98.45	259.4	512.7		224.4	26.5	6.77	17.8	1.92
UG3A	carbonatite tuff	26.47	2.11	6.38		7.15	3.2	0.29	6.63	24.71	0.98	0.72	1.84	3	15.7	250		99.18	262.5	490.5		198	27.4	6.85	17.6	1.85
UG17	carbonatite tuff	16.68	2.1	4.11		8.16	3.54	0.39	7.7	29.79	0.53	0.59	2.68	3.97	16.5	330		96.74	397.4	771.3		251	42	8.9	19.4	2.28

UG23A	carbonatite tuff	20.52	1.77	4.76	7.06	3.46	0.36	4.19	31.46	0.77	0.45	2.41	2.82	17.2	150		97.23	362.1	690.1		282.2	36.1	9.14	21.5	2.43
UG24	carbonatite tuff	19.94	1.8	4.89	6.42	3.93	0.34	4.94	32.47	0.74	0.37	2.35	2.49	17.7	170		98.38	351	665		275.1	34.53	8.91	21	2.47
UG27A	carbonatite tuff	33.66	1.77	8.7	6.61	4.02	0.28	5.3	21.85	1.6	1.17	1.61	2.32	10.2	140		99.09	267.8	518		211.3	27.62	7.13	18.1	2.1
UG27L	carbonatite tuff	37.4	1.66	10.07	6.46	3.55	0.26	4.7	17.95	1.79	1.41	1.63	2.91	8.86	110		98.65	265.5	510		208.7	27.32	7.19	18.3	2.14
**Southern Namibia**																									
MKC 29	calcio carbonatite	1.36	0.01	0.02		2.16	0.74	1.68	51.42	0.01		2.2	0.1			39.69	99.39	239.8	417.5	56.8	216.2	33.2	10.4	26.3	
MKC 52	calcio carbonatite	3.97	0.07	0.33		2.85	0.23	1.31	49.42	0.25	0.07	2.37	0.24			36.68	97.79	242	480.5	53.4	217.1	33.1	9.5	25.6	
MKC 4	magnesio carbonatite	0.13	0.01	0.03		4.34	0.85	20.51	28.01	0.04			0.02			44.99	98.93								
MKC 6	magnesio carbonatite	0.54		0.18		8.46	1.2	19.66	27.97	0.01		0.03	0.17			42.22	100.44								
MKC 7	magnesio carbonatite	0.62	0.02	0.12		7.48	2.22	18.1	29.51	0.04			0.08			42.56	100.75	127.6	163.8	18.4	58.6	6.1	1.5	4	
MKC 9	magnesio carbonatite	0.39	0.02	0.12		5.98	0.98	20.56	27.63	0.02	0.08	0.04	0.04			44.26	100.12	116	159	18.8	60.5	6.5	1.7	4.2	
MKC 18	magnesio carbonatite	0.2		0.01		5.92	0.93	19.2	28.9				0.07			43.87	99.1								
MKC 35	magnesio carbonatite	0.15				5.04	1	19.54	28.8				0.03			43.66	98.22	84.3	104.4	12.4	39.3	4.5	1.2	2.7	
MKC 55A	magnesio carbonatite	0.29		0.1		7.81	1.81	17.87	28.92	0.06			0.11			42.44	99.41								
MKC 62	magnesio carbonatite	0.12		0.06		6.44	1.1	17.36	29.16	0.09			0.14			43.89	98.36								
MKC 34	magnesio carbonatite	0.38	0.07	0.05		3.08	0.75	18	30.24	0.09		1.62	0.14			44.36	98.78	153.8	204.5	25.1	94.3	13.3	4.1	9.9	
MKC 45	magnesio carbonatite	0.13	0.02	0.04		3.67	0.89	17.34	32.42	0.3		3.92	0.09			41.07	99.89	175.2	392	48	204.6	30.5	8.3	21.5	
MKC 46	magnesio carbonatite	1.12	0.06	0.05		7.76	0.79	16.66	30.72	0.46		3.19	0.13			39.26	100.2								
MKC 47	ferro carbonatite	0.39	0.46	0.12		10.38	2.04	14.19	29.92	0.06	0.05		0.08			42.52	100.21	46.1	77.1	7.5	27.6	3.4	0.9	2.4	
MKC 48	ferro carbonatite	3.02	0.01	0.93		15.13	1.83	10.33	28.38	0.24	0.04		0.33			38.63	98.87								
MKC 15	ferro carbonatite dyke	2.57		0.03		22.81	7.74	1.13	33.06	0.35	0.08	0.01	1.16			27.99	96.93	1546	3377	519.8	1932	203	47.6	80	
MKC 16	ferro carbonatite dyke	0.8		0.01		24.15	6.79	6.04	29.07	0.2			0.29			29.23	96.58	533	1136	174.8	661.6	75.5	20.7	38.2	
MKC 17	ferro carbonatite dyke	0.63		0.03		18.98	5.57	6.84	29.72	0.07		0.01	0.18			32.05	94.08								
MKC 20A	ferro carbonatite dyke	1.68		0.61		20.27	1.63	5.53	26.94	0.07	0.04	0.2	0.28			33.54	90.79								
MKC 20B	ferro carbonatite dyke	1.35		0.41		14.79	1.34	6.12	25.4	0.13		0.21	0.36			32.43	82.54								
MKC 27	ferro carbonatite dyke	0.24		0.05		11.18	5.09	11.87	28.95	0.07		0.69	0.03			38.91	97.08	1190	1950	245.7	833	76.1	17.4	29.6	
**Swartbooisdrif, Namibia**																									
PB24	carbonatite	3.23	0.01	0.69	19.47		2.16	8.05	27.36	0.42	0.01	0.02					99.1	44.1	101	12.3	47.3	8.79	2.85	7.38	1.06
PB24a	carbonatite	2.99	0.01	0.67	19.29		2.23	8.11	27.58	0.47	0	0.01					99.37								
PB27	carbonatite	10.37	0.02	2.78	17.55		1.97	7.5	24.04	1.68	0.01	0.03					99.02	55	123	16	61.4	11.65	4.06	10.03	1.6
PB28	carbonatite	18.33	0.18	5.42	18.36		1.72	6.15	19.33	3.19	0.04	0.03					99.35	129	214	23.6	79	11.88	3.69	9.27	1.37
PB29	carbonatite	3.14	0.01	0.27	19.19		2.24	8.43	26.96	0.24	0.01	0.02					98.77	55.1	126	16.1	60.7	11.43	3.71	9.65	1.43
**Vuoriyarvi,Kola**																									
BR 18	carbonatite	7.01	0.29	0.81	2.35	1.62	0.17	4.17	45.38	0.3	0.57	4.36					100.37	368	793	103	391	63	19.4	51	
BR 30	carbonatite	2.23	0.08	0.03	0.54	1.37	0.25	2.43	49.39	0.32	0.25	5.79					99.73	482	996	125	449	60	17.3	42	
BR 35	carbonatite	2.49	0.12	0.4	1.01	0.86	0.1	2.57	52.09	0.09	0.23	1.74					100.4	300	609	73	269	39	11.8	30	
BR 64	carbonatite	1.25	0.08	0.31	2.57	2.05	0.12	1.84	52.19	0.11	0.26	3.41					100.84	147	306	39	146	23	6.6	17.3	
**Turiy Massif**																									
C.49.40	carbonatite	4.98	0.2	1.21	7.91		0.16	6.52	39.45	0.03	1.26	6.97				28.5	97.19	54	111	11.1	47.9	8.2	2.35	6.3	0.81
C.TL.344	carbonatite	4.81	0.75	0.26	12.57		0.14	2.39	42.58	0.21	0.14	4.75				28.6	97.2	74	144	14.8	63.1	10	2.96	8.2	0.95
C.DC	dolomite carbonatite	0	0.01	0.01	1.87		0.31	9.34	42.84	0	0.01	2.2				43.1	99.69	89	159	14.4	55.4	8.3	2.32	6.4	0.7
S.W58	carbonatite	0.71	0.17	0.15	5.07		0.64	4.8	41.63	0.02	0.12	3.37				38.8	95.48	1119	1944	149.6	511.2	61.6	15.52	45.3	5.1
**Southern Italy**																									
VM5	Lapilli tuff	12.9	0.23	4.61	2.91		0.22	1.7	40.4	0.39	0.69	1.24		28.9		32.2	99.1	373	592	56	168	21.6	4.6	14.1	1.72
VM6	Ash tuff	12.8	0.32	4.45	5.84		0.38	2.53	39	0.33	0.44	2.36		25.5		29.1	99.8	697	1203	113	357	47	10	30.5	3.7
VM7	Rounded lapilli	18	0.5	6.1	6.59		0.35	2.77	36.6	0.71	0.39	2.21		20.8		24.5	100.4	619	1051	100	318	43	9.2	28.3	3.5
VM8	Upper lava flow	16.3	0.46	5.41	6.51		0.35	2.96	40.2	0.47	0.17	2.18		17.4		22.4	99.4	604	1027	98.9	313	42	9.2	28.2	3.4
VM9	Feeder dike	17.2	0.47	5.57	6.73		0.36	3.06	39.5	0.47	0.19	2.23		18.3		21.9	99.3	621	1054	102	319	43	9.1	28.9	3.4
SVK116	Massive alvikite	18.7	0.51	4.78	4.55		0.07	10.9	36.2	0.55	0.72	0.42		16.9		22.94	100.6	94	211	25.6	102	18.6	3.3	12.1	1.44
**Germany**																									
H31	carbonatite	1.28	0.15	0.45	2.47		0.37	0.75	51.1	0.09	0.05	1.57	2.15	38.95			98.38	250	460		110				
KB12	carbonatite	0.48	0.03	0.17	0.94		0.35	0.33	52.9	0.06	0.03	1.21	0.54	40.51			98.2	240	430		90				
219	carbonatite	5.09	0.44	1.31	5.98		0.57	2.29	44.75	0.1	0.05	2.93	5.3	29			97.81	575	1055		275				
5/19N	carbonatite	1.68	0.05	0.53	2.75		0.46	0.8	50.1	0.02	0.03	1.61	2.95	36.25			97.23	630	1310		280				
5/19Z	carbonatite	1.09	0	0.17	0.91		0.46	0.31	52.2	0	0	1.23		41.1			97.47	660	1240		260				
5/12	carbonatite	4.93	0.37	1.06	5.28		0.55	1.82	44.45	0.1	0.6	2.88	1.46	33.04			96.54	540	1110		280				
6/20	carbonatite	2.07	0.24	0.88	5.04		0.66	1.1	47.9	0.01	0.02	2.14	0.51	36.39			96.96	610	1010		260				
11/4	carbonatite	1.17	0.04	0.15	1.26		1.16	0.54	49.9	0.18	0.11	0.99		40.8			96.3	560	900		110				
425	carbonatite	1.37	0.05	0.25	3.18		0.42	3.05	48.6	0.1	0.03	2.87	0.47	36.81			97.2	448	1101		257				

Dy	Ho	Er	Tm	Yb	Y	Lu	Cr	Ni	Rb	Sr	Ba	Th	U	Pb	Nb	Ta	Zr	Hf	Sc	V	Cu	Zn	Co	Total REE	Reference
244	24.7	49	2.2	12.6	580	1.62	4.72	25.5	2.62	2961	43360	17.5	1.54	255	109	0.37	20.1	1.46	12.5	23.4	44.3	406	6.84	187294	Sun et al., 2013
104	16.8	42.8	4.7	29.2	278	4.03	39.7	46.3	11.7	2862	1928	484	3.29	55.5	122	0.91	44.7	1.73	33.2	53.3	4.72	207	11.8	16979	Sun et al., 2013
19.5	3.29	8.17	0.87	5.26	90.5	0.71	3.94	10.8	0.4	1577	142	5.79	18.5	58.8	173	27.7	3.89	0.25	9.85	0.64	1.6	36.1	3.81	1564	Sun et al., 2013
22.2	3.33	7.74	0.84	4.24	79	0.5			1.06	14324	8794	1.43	0.09		1.38	0.1	5.33	0.2							Yang et al., 2011
54.7	8.83	17.9	1.72	8.03	260	0.95			67.3	2930	2039	145	1.35		120	1.8	197	9.56							Yang et al., 2011
261	21.7	27.8	2.6	16.8	506	2.06			6.64	5046	1573	1669	4.02		16.6	0.21	2.32	0.66							Yang et al., 2011
107	10.8	13.2	1.35	8.49	281	1.19			2.89	7234	42954	884	2.61		5.7	0.14	2.01	0.29							Yang et al., 2011
27.9	15.5	24.8	8.1	2.8	190	0.7	20	35		1500	3295	181		526	285				56	86	115	250	26	14633	Yang et al., 2009
341	41.2	45.5	11.5	52	179	8.1	15	21		1524	5623	254		700	112				45	89	38	236	14	150459	Yang et al., 2009
352	45.3	265.8	13.2	35.5	280	11.2	16	23		5632	27786	395		756	208				48	92	126	589	12	110301	Yang et al., 2009
565.6	39.7	79.5	7	36	285	5.1	2	22		10925	43917	906			723				40	11	55	506	19	199154	Yang et al., 2009
30.9	4.62	4.87	1.59	7.84	137	0.98	45.1	48.4	28.1	3008	1525	170	22.1	17.1	92.8	2.51	30	0.61		125		137	23.4		Ying et al., 2004
25.7	3.62	3.62	1.1	5.61	105	0.73	85.5	85.8	45.9	4315	3483	52.7	17.9	41.9	37.4	1.4	425	8.96		149		133	31.2		Ying et al., 2004
35.3	4.9	5.42	1.61	7.87	149	0.97	6.4	15.7	4.6	5366	18072	74.5	11.6	30.5	26.4	0.09	58.9	0.34		86.4		38.1	5.6		Ying et al., 2004
39.9	5.37	5.53	1.51	7.54	165	0.95	3.7	11.7	6.4	4911	12971	98.7	8.41	14.6	100	2.82	266	2.85		108		41	2.5		Ying et al., 2004
10.8	1.48	1.53	0.47	2.54	40.6	0.33	156	122	75	2278	1979	18.8	2.07	9.72	20.7	1	593	13.6		121		83.9	29.8		Ying et al., 2004
13.5	2.08	2.32	0.8	4.43	67.3	0.55	80.3	65.6	231	1767	596	15.4	4.34	5.01	87.2	0.72	247	7.01		119		71.7	18.6		Ying et al., 2004
24.8	3.55	8.76	0.89	4.9	91.3	0.61	15.6	11.7	29.9	2535	6878	80.1	9.17	13.6	75.1	1.1	479	7.88		50.1		16.4	8.6		Ying et al., 2004
23.8	3.33	8.22	0.79	4.5	90.9	0.58	10.6	12.1	14.9	1765	1423	73.2	9.51	41.1	94.6	2.21	596	8.94		65.8		17.9	11.8		Ying et al., 2004
23.2	3.24	7.96	0.78	4.44	86	0.58	11.2	14.4	16.3	1645	574	70.3	7.62	61.7	75.4	1.56	432	7.04		85.6		23.3	9.8		Ying et al., 2004
28.3	4.06	9.89	0.98	5.69	104	0.78	7.3	27.3	26.7	927	9443	123	5.94	12.9	62	3.07	155	3.11		84.5		219	13.8		Ying et al., 2004
21.4	3.09	7.4	0.74	4.07	77.1	0.52	113	91.4	66.9	2731	3066	53.7	21.5	6.35	147	2.12	574	10.11		109		138	24.5		Ying et al., 2004
51.4	6.51	6.7	1.79	8.81	185	1.11	10.9	17.4	25.6	2721	16737	61	12.8	21.1	111	5.5	161	2.9		92.1		91.2	12.1		Ying et al., 2004
20.1	2.71	6.47	0.59	3.29	69.1	0.42	7.1	15.1	14.5	2437	6945	66.6	9.78	16.2	92.9	1.42	154	2.55		40.8		109	13.6		Ying et al., 2004
							13.49	5		119581	131033	406			5		5							89519	Caster, 2008
							12.42	12		21043	224987	241			31		25							118270	Caster, 2008
							11.49			17747	296459			92.8										60949	Caster, 2008
							16.73			5071	121808			92.8										126251	Caster, 2008
							29.78			17747	15226			464										11247	Caster, 2008
							17.54			59157	89565			6589										59044	Caster, 2008
							14.01			101412	204208			3619										80358	Caster, 2008
							18.50			45635	135243			186										62128	Caster, 2008
							17.61			39720	176443			5939										22493	Caster, 2008
4.26	0.64	1.49	0.22	1.43	15.3	0.25				5940	263	45.4	0.45	8.94			3.20	<0.5						224	Andrade et al.,1999
7.30	1.07	2.26	0.29	1.57	24.9	0.22				8140	643	82.5	0.30	74.00			<13	<0.5						289	Andrade et al.,1999
3.04	0.50	1.13	0.18	1.06	12.3	0.17				9620	179	46.7	<0.3	<10			29.80	0.23						273	Andrade et al.,1999
3.12	0.52	1.32	0.21	1.44	12.8	0.23				2770	495	36.6	0.25	40.60			5.90	<0.5						408	Andrade et al.,1999
3.55	0.65	1.86	0.27	2.06	17.7	0.35				4770	428	21.9	0.38	20.50			10.40	<0.5						646	Andrade et al.,1999
6.87		4.59		1.89	89	0.36	9	9	20	4848	4432	20			222		177				51	19		931	Srivastava，1997
7.42		4.12		3.05	120	0.51	11	16	15	3296	3305	15			236		244				55	8		665	Srivastava，1997
10.22		6		3.84	111	0.56	11	16	19	4721	2933	15			341		244				55	8		1355	Srivastava，1997
22.47		11.8		8.39	92	0.59			7	4009	2970				243									3473	Srivastava，1997
31		16.01		9.16	113	1.38	11	14	10	5082	3601	6			340		99				63	16		2937	Srivastava，1997
23.35		10.82		6.74	87	1.18	11	14	18	2003	1886	6			311		99				63	16		3832	Srivastava，1997
159		56.7		31.2	961				1	3629	1879				868		165								Le Roex and Lanyon , 1998
6.51		1.31		0	19				114	855	12317				225		27								Le Roex and Lanyon , 1998
58		15.3		0	223				145	24686	1200				975		242								Le Roex and Lanyon , 1998
45.3		8.49			110				0	36940	3763				23		5								Le Roex and Lanyon , 1998
14.9		4.06			83				0	42685	2804				706		800								Le Roex and Lanyon , 1998
67.9		16.2			276				0	33874	3801				1005		785								Le Roex and Lanyon , 1998
70.2		19.5		8.28	285				0	23967	2089				768		573								Le Roex and Lanyon , 1998

58.7		17.4		7.92	275				33	16738	2235				1440		479								Le Roex and Lanyon , 1998
8.73		4.7		4.75	64				60	9866	2514				1961		50								Le Roex and Lanyon , 1998
38.3		13.4		5.57	194				355	16299	8274				355		55								Le Roex and Lanyon , 1998
					29		<10	21	34	33890	4400				64		384			15		38			Simonetti and Bell,1994
					134		<10	30	<10	2090	220				245		23			39		110			Simonetti and Bell,1994
					190		<10	29	<10	1530	2020				508		144			60		50			Simonetti and Bell,1994
					121		<10	25	<10	9920	3920				53		68			97		1070			Simonetti and Bell,1994
					25		<10	21	<10	2790	539				24940		522			884		215			Simonetti and Bell,1994
					168		<10	29	<10	8280	667				126		68			63		83			Simonetti and Bell,1994
					133		<10	62	<10	6810	1240				150		81			122		807			Simonetti and Bell,1994
					561		<10	69	<10	3500	151400				19		208			50		2190			Simonetti and Bell,1994
				126	50				80	3510		4	15	30	80		60				30	60		1447	Secher and Larsen, 1980
				3.15	50				120	1660		4	15	30	310		60				30	60		1309	Secher and Larsen, 1980
					110	0.32			50	1390		4	15	30	380		60				30	60		1530	Secher and Larsen, 1980
					30	1.24			90	870		73	8	30	160		140				30	100		1171	Secher and Larsen, 1980
				0.72		0.13				1180		73	8	30	>1%		140				30	100		1058	Secher and Larsen, 1980
				0.5	100	0.23			70	1440		73	8	30	230		140				30	100		1115	Secher and Larsen, 1980
				2.43		0.31				640					>15%		83				2.5			1087	Secher and Larsen, 1980
				1.71	96					3400		4	15	30	1600		60				30	60		1101	Secher and Larsen, 1980
					62		22	4	37	13449	1343	13			416		184		40	96	27	76	20	1392	Zaitsev et al., 1998
					44		40	6	25	12307	1268	10			515		135		8	112	76	44	18	1570	Zaitsev et al., 1998
					113			24		63819	13102	127							56		39	57	23	66374	Zaitsev et al., 1998
					137		15	18		73004	10205	103			10		5		28	14	28	120	14	86292	Zaitsev et al., 1998
					98		10	20		20495	11380	44			15				21	25	47	995	19	49930	Zaitsev et al., 1998
					52			24		16174	10990	17			28				32		84	2354	bd	60449	Zaitsev et al., 1998
					43		286	15	5	5533	28374	84			112		7		19	201	45	1850	40	9363	Zaitsev et al., 1998
					102		4	16	9	7017	1280	76			86		20		21	156		3073	4	12932	Zaitsev et al., 1998
20.2	2.9	7.5	0.8	4	90	0.5		60	16.7	8550	1820	190	6.9	10	55	0.5	26.5	1	4.9	426	533	109	131	4423	Shivdasan et al., 2003
29.5	4	10.2	1.1	5.6	127	0.5		31	19.9	6540	473	501	11.2	7	94	0.5	45.5	1	6	758	396	311	168	7232	Shivdasan et al., 2003
25.6	4	9.4	1	4.7	109	0.5		8	7.1	>10000	1150	394	23.2	7	109	0.9	44.6	1	7.6	131	47	46	22.3	6645	Shivdasan et al., 2003
10.1	1.6	4	0.5	3.2	46	0.4		37	9.5	7180	4210	168	1.9	11	89	0.5	2.8	1	0.6	564	121	259	100	2703	Shivdasan et al., 2003
24.2	3.9	10.7	1.4	10.4	106	1.3		6	0.4	>10000	>10000	190	1.6	50	107	0.5	3.2	1	5.1	9	9	990	3	3855	Shivdasan et al., 2003
13.1	2	5.8	0.7	5	65	0.6		11	5.4	>10000	2550	212	14.6	48	450	5.1	191	2	6	304	12	84	10.4	2578	Shivdasan et al., 2003
9.5	1.5	4.1	0.5	2.9	46	0.3		5	1.1	>10000	2880	34	0.7	10	5	0.5	2.3	1	1.4	6	7	15	0.9	1939	Shivdasan et al., 2003
36.8	5.8	15.8	1.5	8.9	171	0.8		5	0.5	9870	3520	175	13.9	7	129	0.5	43.6	1	3.6	27	8	27	2.5	6403	Shivdasan et al., 2003
4.7	0.91	1.86		1.4	22.86	0.25			5.88	3115	317	0.46	<0.1	0.99	5.21	0.75	42.5	0.59							Verhulst et al., 2000
4.89	0.87	1.8		1.33	22.6	0.2			4.9	3045	246	0.86	<0.1	<0.5	2.56	0.47	105	1.62							Verhulst et al., 2000
3.23	0.62	1.26		0.95	15.3	0.18			3.13	2406	208	0.05	<0.1	<0.5	6.86	2.11	282	5.6							Verhulst et al., 2000
6.94	1.15	2.04		1.34	29	0.2			14.3	7036	531	5.02	30.4	<0.5	76	27.6	144	3.32							Verhulst et al., 2000
1.3	0.23	0.44		0.3	5.35	0.03			6.6	3221	46	1.2	5.07	<0.5	31	6.42	3.87	<0.1							Verhulst et al., 2000
1.01	0.16	0.26		0.17	3.9	<0.02			10.4	2778	72	5.01	38.6	<0.5	84	35.2	3.07	<0.1							Verhulst et al., 2000
17.27	2.81	7.31	1.07	4.78	74.3	0.42			2.9	8735	175	44.8	3.3		251	9.4	45.9	2.9						872	Can comin-chiaramonti,et al, 2001
67.28	12.33	24.85	2.83	10.22	121	1.53			4.3	2134	3798	19.9	3		384	18.7	123	7.7						1340	Can comin-chiaramonti,et al, 2001
39.92	6.7	16.04	2.57	8.49	191.8	2.2			6.8	8758	1134	81.5	5		22.9	0.8	22.9	0.2						1788	Can comin-chiaramonti,et al, 2001
85.85	9.7	26.49	3.8	22.62	225.2	3.34			10.2	1053	449	146.6	30.2		95.1	6.3	182.8	3.7						12228	Can comin-chiaramonti,et al, 2001
30.15	5.13	10.36	1.73	11.29	96.4	1.8			1.6	3693	8378	811.2	71		315.2	11.8	122.9	2.4						3119	Can comin-chiaramonti,et al, 2001
90.03	18.23	46.97	6.92	34.09	673	5.71			189.4	4652	2226	1178	122.3		25.1	1.4	177.9	4.9						4509	Can comin-chiaramonti,et al, 2001
			0.61	3.76	53	0.51	49	28		5830	2717	64	14.4		509	24.4	667	8.9	13.5	291		179	30.2	1803	Eby et al., 2009
			0.45	2.72	38	0.37	142	73	10	4387	2441	30.7	10.8		381	14.5	457	7	15.4	321		122	33.2	1252	Eby et al., 2009
			0.37	2.31	33	0.32	73	38	15	3578	1974	32.2	8.9	2	309	14.3	452	7.2	13.2	174		97	27.6	1106	Eby et al., 2009
			0.32	2.11	29	0.27	78	35	16	3460	2261	29.3	7.8		274	11.5	415	6.1	11.3	150		104	25	1052	Eby et al., 2009
			0.39	2.27	33	0.31	99	53	12	3332	1786	32	8.2	4	295	14.1	453	7.2	16.1	222		109	30	1008	Eby et al., 2009
				3.59	50	0.46	63	36	6	3904	2728	40.6	11.8	7	435	16.2	617	6.5	14.3	228		128	32.6	1496	Eby et al., 2009

			0.5	3	43	0.41	14	24		4215	2049	54.7	11.5	11	394	14.86	483	6.3	12.3	154		135	28.6	1407	Eby et al., 2009
			0.53	2.78	40	0.41	15	25		4125	2552	54.2	11.2	12	372	15.45	465	6.3	13.8	173		118	28.9	1362	Eby et al., 2009
			0.45	2.86	36	0.41	27	36	23	3420	2621	41.5	7.7	10	253	11.35	420	7	17	219		116	29.4	1056	Eby et al., 2009
			0.47	2.96	38	0.4	35	40	30	3324	2754	40.1	7.2	9	241	10.58	425	7	17.1	207		118	29.2	1043	Eby et al., 2009
20.6	4	9.6		6.8	92		79	8		6922	455	28	5	8	1982		71			32	65	19	11	1041	Smithies and Marsh,1998
17.5	3	8		5.2	70	0.7	<5	7	7	2745	345	25	30	13	950		78			46	33	24	1	1096	Smithies and Marsh,1998
					4		10	4		6103	241	3	1	13	716		33			23	67	45	7		Smithies and Marsh,1998
					19		30	1	2	2195	702	18	9	21	1008		11			42	26	50	3		Smithies and Marsh,1998
	0.7	1.5		0.7	7		22	4		5331	62	8	2	31	785		49			34	61	130	3	383	Smithies and Marsh,1998
	0.5	1.1		0.6	3		<6	6		7708	132	2	1	15	91		6			24	81	82	16	369	Smithies and Marsh,1998
							42	4		7618	499	3	2	112	17		48			34	72	214	3	0	Smithies and Marsh,1998
	0.4	0.9		0.5			54	7		7397	485	6	7	5	106		2			29	72	39	6	251	Smithies and Marsh,1998
					1		7	3		6015	213	15		35	551		12			<5	69	108	5		Smithies and Marsh,1998
					2		<7			7663	65	13		10	1		58			<5	86	28			Smithies and Marsh,1998
5.7	1	2.1		1.1	16		37	2		4715	79	4		1	611		50			<10	45	43		515	Smithies and Marsh,1998
11.4	1.7	4.4		1.7	30	0.2	20	6		5192	<15	7		11	2029					<5	62	47		900	Smithies and Marsh,1998
					25		<10	6		4923	40	86		4	1737					43	57	38		0	Smithies and Marsh,1998
2.7	0.5	1.9		2	13	0.3	40	5	2	1268	<21	137		12	378					45	19	75		172	Smithies and Marsh,1998
					51		47	15		7513	141	196		19	324					30	89	7184			Smithies and Marsh,1998
	6	10.1		2.9	44		<10	8		1575	203	412		32	7					<10	76	3883		7724	Smithies and Marsh,1998
	3.5	6.5		2.7	56		53	13		4632	510	712		27	190					<10	58	1488		2652	Smithies and Marsh,1998
							19	16		8363	584	1015		205						<10	83	164			Smithies and Marsh,1998
							<10	15	10	15579	16303	9		127						<10	146	244			Smithies and Marsh,1998
							<10	29		43778	26229			160						<11	385	832			Smithies and Marsh,1998
	2.8	4.7		1.4	11		30	8		7377	5390			20	5					<10	78			4351	Smithies and Marsh,1998
5.68	1.02	2.66	0.41	2.48	28.5	0.38	27	37	0.22	5105	146	0.65	0.05	16.6	15.2	0.03	0.98	0.1	12.7	37	8	101		237	Thompson etal ,2002
																									Thompson etal ,2002
8.37	1.63	4	0.69	4.04	44.1	0.66	58	40	0.49	6085	185	1.99	0.27	19.6	120	0.05	3.2	0.11	11.9	69	2.5	149		302	Thompson etal ,2002
7.15	1.34	3.33	0.57	3.21	36.5	0.52	38	27	0.44	4487	169	4.39	0.19	18.9	136	0.18	2.79	0.09	16.3	25	7.1	164		488	Thompson etal ,2002
7.46	1.4	3.52	0.57	3.43	38.8	0.52	17	39	0.18	5062	156	0.51	0.02	20.4	22.3	0.06	2.08	0.1	12.3		3.1	205		301	Thompson etal ,2002
30	4.9	10.4		6.2	121	0.8									53	6.7								2163	Brassinnesa et al., 2005
19.8	3.1	6.1		3.7	79	0.5									1300	1.3								3617	Brassinnesa et al., 2005
16	2.5	5.1		3	67	0.3									47	6.7								1672	Brassinnesa et al., 2005
9.1	1.4	3.1		1.9	41	0.2									10	0.6								870	Brassinnesa et al., 2005
3.91	0.61	1.44	0.13	0.8	16	0.13			32	4580	347	1.35	0.14		8	1.46	13			92	20	36	49.8	249	Dun worth and Bell., 1998
4.74	0.78	1.85	0.19	0.76	20	0.16			3	3284	244	6.53	0.21		11	1.02	181			123	94	59	30.6	326	Dun worth and Bell., 1998
3.21	0.54	1.37	0.13	0.69	14	0.12			3	7123	458	1.7	0.32		3	0.15	6			6	6	12	8.8	342	Dun worth and Bell., 1998
23.42	3.96	9.48	0.84	3.57	110	0.51			2	22397	1576	102.02	14.91	163	391	3.03	164			110	19	242	6.5	3893	Dun worth and Bell., 1998
8.6	1.43	3.4	0.5	2.89	47	0.39	12	26	92	0.98	0.4	36	27.3	47	207	5.3	187		7	151	19		13		D'Orazio et al., 2013
17.9	3.01	7	1.02	6	99	0.77	7	19	33	1.11	0.85	92	63	88	389	11.2	169		5	303	28		17		D'Orazio et al., 2013
16.5	2.8	6.6	0.96	5.4	91	0.72	7	20	11.1	0.74	0.72	85	43	72	320	9.7	175		7	373	43		19		D'Orazio et al., 2013
16.1	2.88	6.8	1.03	5.6	87	0.76	7	22	21.2	1.05	0.64	85	56	70	332	10.2	210		7	316	40		19		D'Orazio et al., 2013
16.6	2.76	6.7	0.98	5.6	86	0.76	10	21	8.2	0.71	0.15	91	56	71	337	10.2	175		8	393	39		20		D'Orazio et al., 2013
6.4	0.97	2.13	0.26	1.39	30	0.2	542	351	42	0.15	0.09	39	8.8	21	13.2	0.56	334		11	48			36		D'Orazio et al., 2013
					40					3980	900	11	< 5		470		50								Hubberten et al., 1988
					30					6230	1190	<5	<5		510		60								Hubberten et al., 1988
					105					5950	1250	60	7		730		340								Hubberten et al., 1988
					80					7820	880	20	12		1200		150								Hubberten et al., 1988
					80					7990	960	<5	11		890		40								Hubberten et al., 1988
					115					5970	4560	60	10		285		285								Hubberten et al., 1988
					130					7880	1020	<5	<5		610		60								Hubberten et al., 1988
					0					8810	6220	<5	5		630		40								Hubberten et al., 1988
					75					9013	654	33	19		2408		179								Hubberten et al., 1988

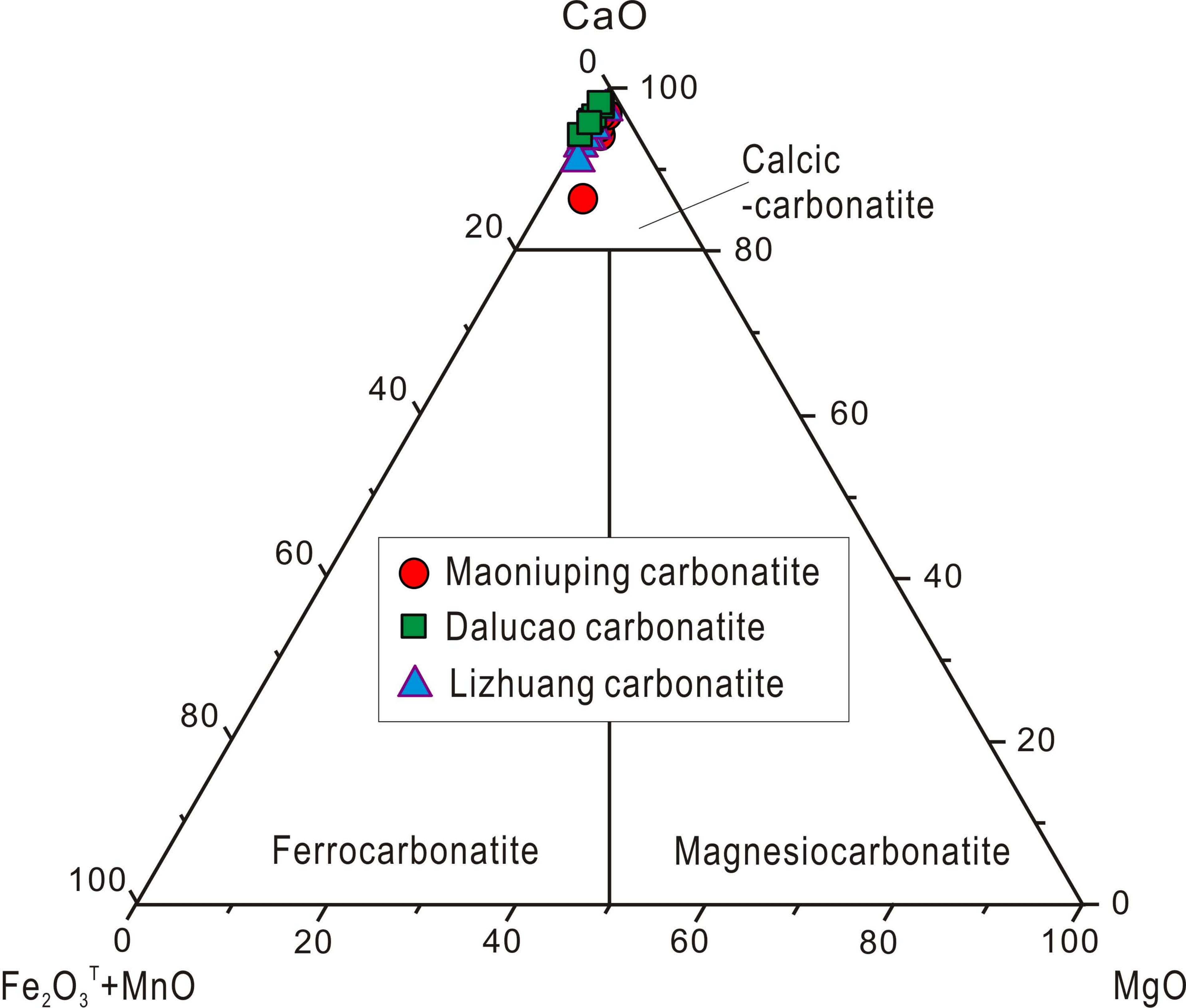
CaO
0
100
Calcic
-carbonatite
20
80
40
60
60
40
80
20
100
0
Maoniuping carbonatite
Dalucao carbonatite
Lizhuang carbonatite
Ferrocarbonatite
Magnesiocarbonatite
0
20
40
60
80
100
Fe2O3T+MnO
MgO

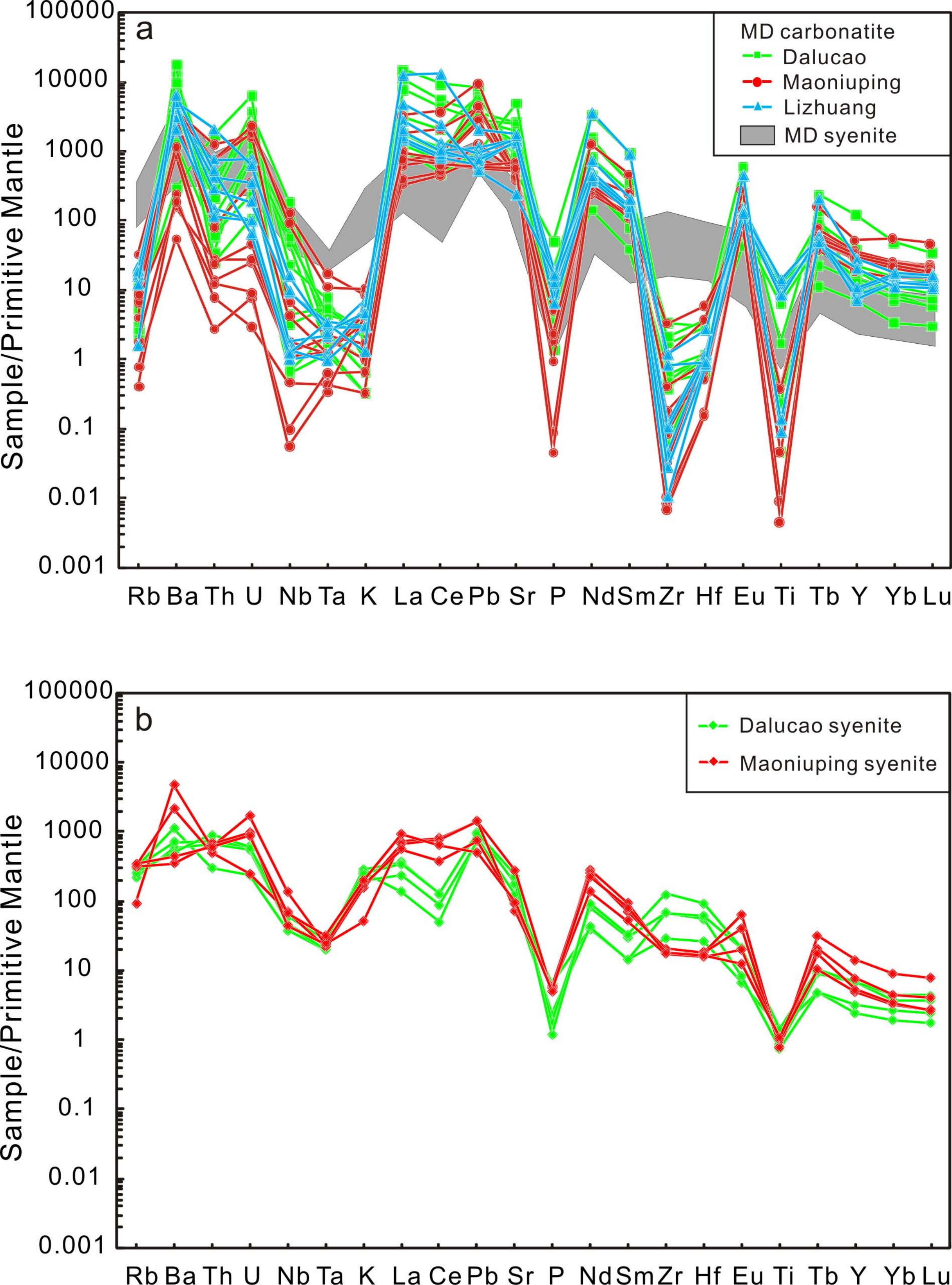
a
MD carbonatite
Dalucao
Maoniuping
Lizhuang
MD syenite
Sample/Primitive Mantle
100000
10000
1000
100
10
1
0.1
0.01
0.001
Rb Ba Th U Nb Ta K La Ce Pb Sr P Nd Sm Zr Hf Eu Ti Tb Y Yb Lu
b
Dalucao syenite
Maoniuping syenite
Sample/Primitive Mantle
100000
10000
1000
100
10
1
0.1
0.01
0.001
Rb Ba Th U Nb Ta K La Ce Pb Sr P Nd Sm Zr Hf Eu Ti Tb Y Yb Lu

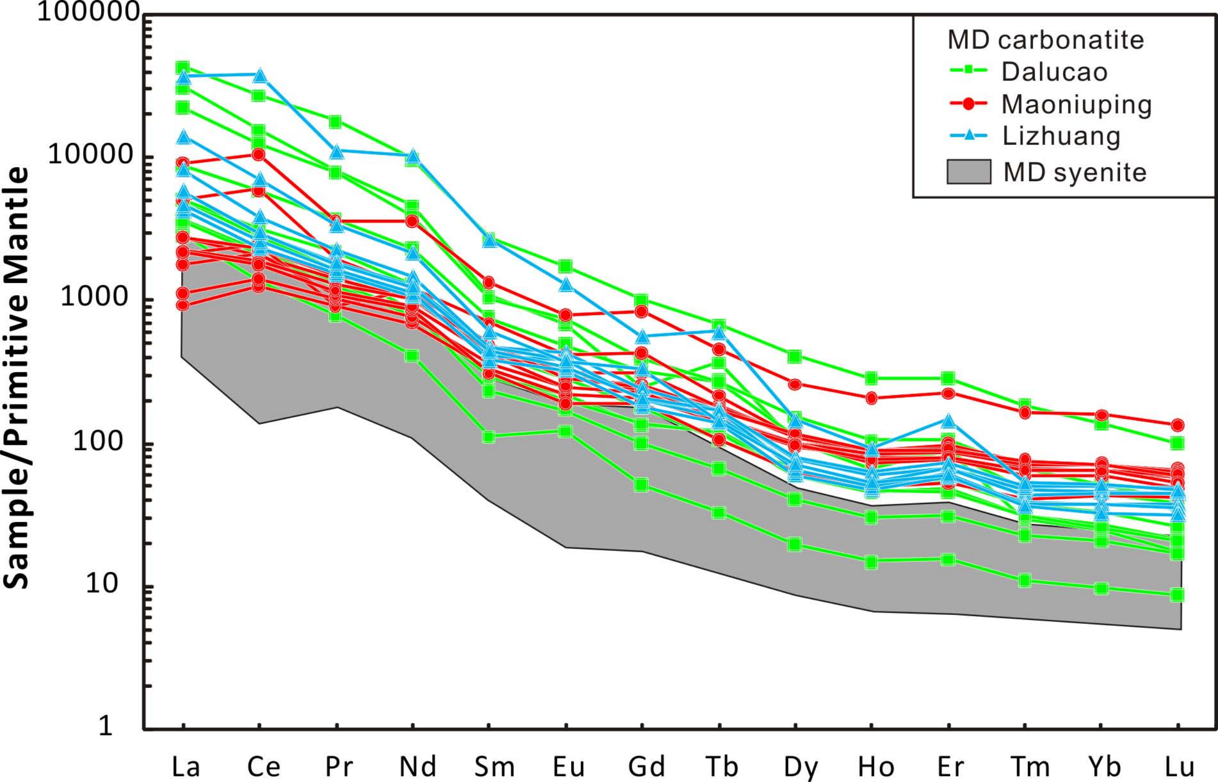

MD carbonatite
Dalucao
Maoniuping
Lizhuang
MD syenite
Sample/Primitive Mantle
100000
10000
1000
100
10
1
La
Ce
Pr
Nd
Sm
Eu
Gd
Tb
Dy
Ho
Er
Tm
Yb
Lu

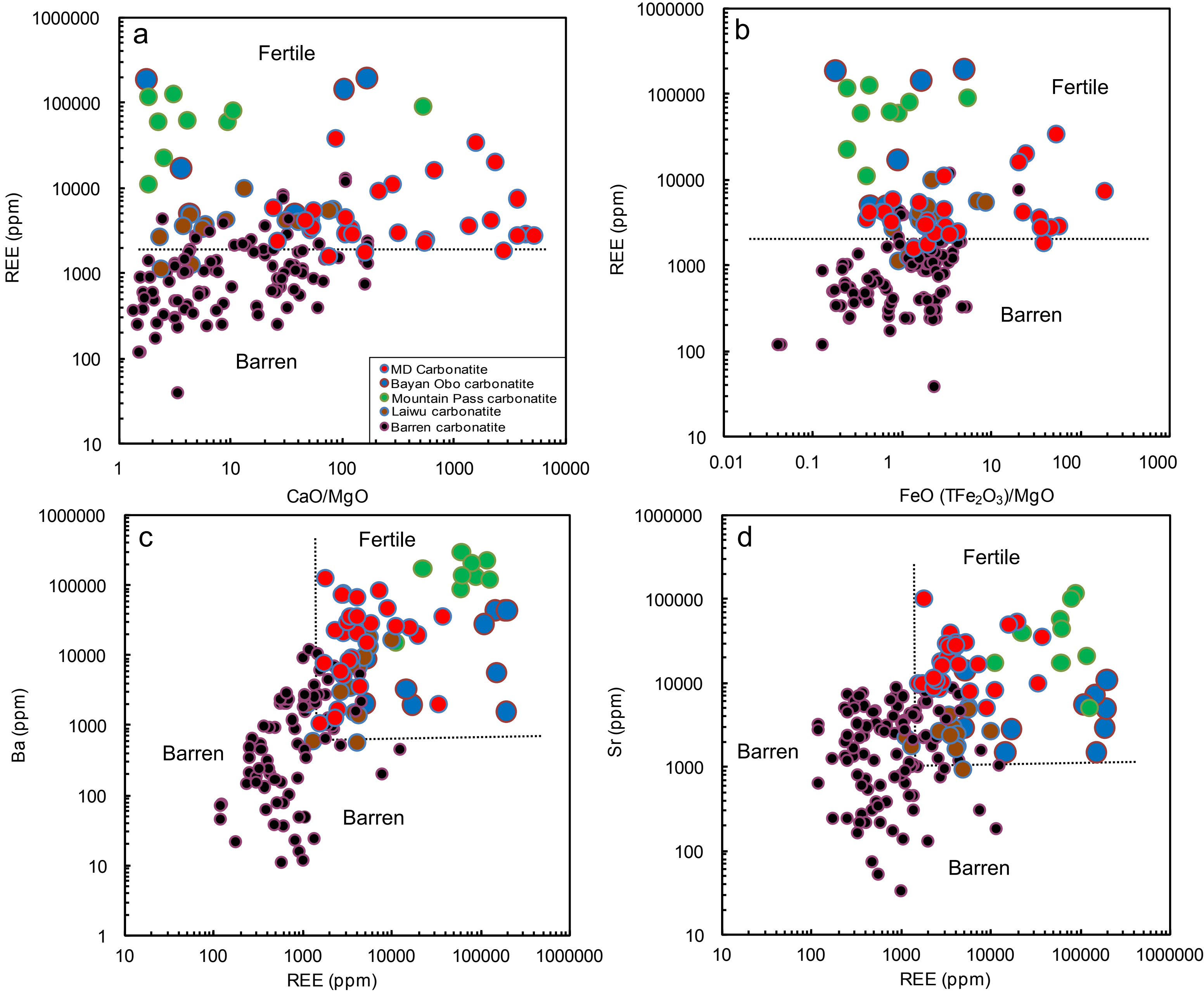

a
Fertile
Barren
REE (ppm)
CaO/MgO
MD Carbonatite
Bayan Obo carbonatite
Mountain Pass carbonatite
Laiwu carbonatite
Barren carbonatite
b
Fertile
Barren
REE (ppm)
FeO (TFe2O3)/MgO
c
Fertile
Barren
Barren
Ba (ppm)
REE (ppm)
d
Fertile
Barren
Barren
Sr (ppm)
REE (ppm)

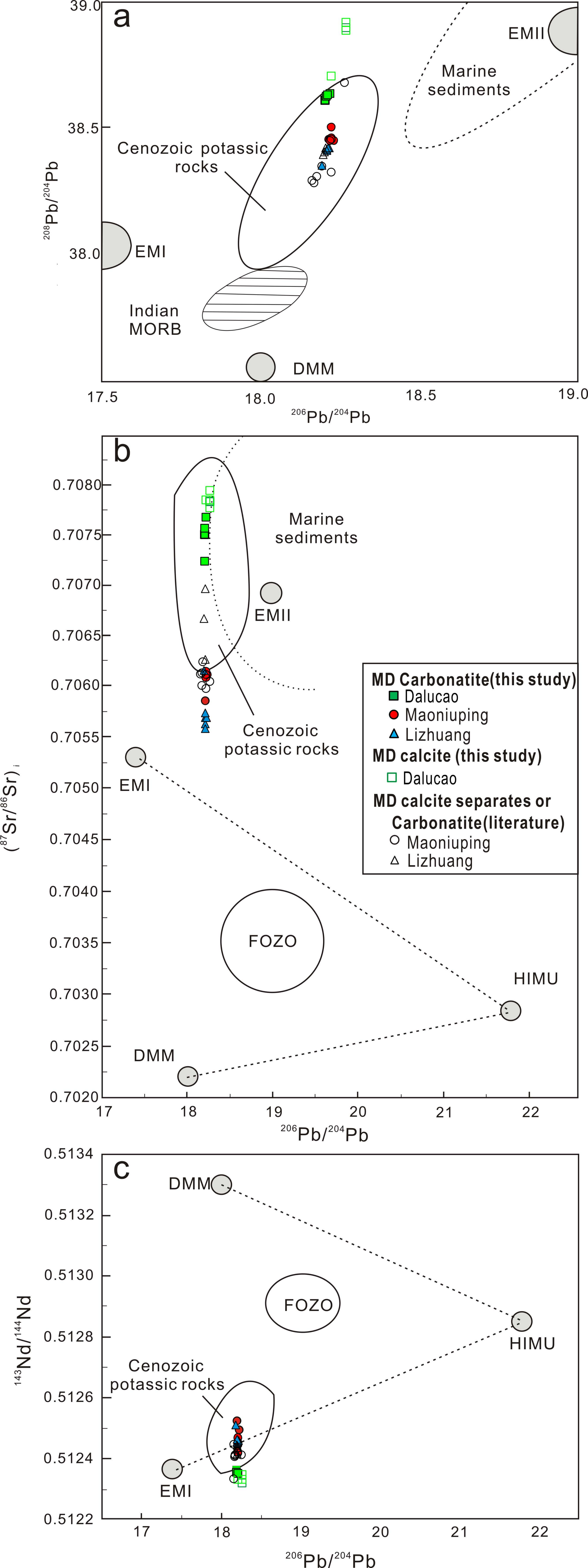
a
EMII
Marine sediments
Cenozoic potassic rocks
EMI
Indian MORB
DMM
$^{208}Pb/^{204}Pb$
$^{206}Pb/^{204}Pb$
b
Marine sediments
EMII
Cenozoic potassic rocks
EMI
FOZO
HIMU
DMM
MD Carbonatite(this study)
Dalucao
Maoniuping
Lizhuang
MD calcite (this study)
Dalucao
MD calcite separates or Carbonatite(literature)
Maoniuping
Lizhuang
$(^{87}Sr/^{86}Sr)_i$
$^{206}Pb/^{204}Pb$
c
DMM
FOZO
HIMU
Cenozoic potassic rocks
EMI
$^{143}Nd/^{144}Nd$
$^{206}Pb/^{204}Pb$

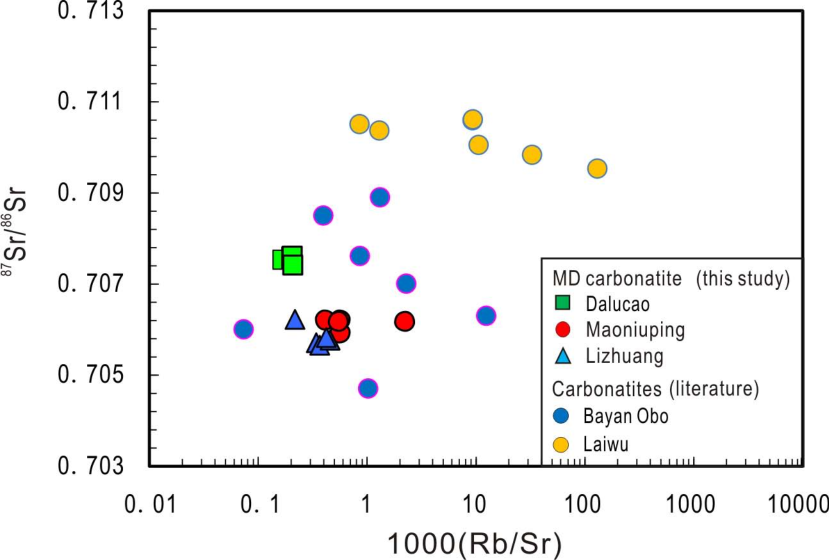

0. 713
0. 711
0. 709
0. 707
0. 705
0. 703
87Sr/86Sr
0. 01
0. 1
1
10
100
1000
10000
1000(Rb/Sr)
MD carbonatite (this study)
Dalucao
Maoniuping
Lizhuang
Carbonatites (literature)
Bayan Obo
Laiwu
